# Cellulosomics of the cellulolytic thermophile *Clostridium clariflavum*

**DOI:** 10.1186/1754-6834-7-100

**Published:** 2014-07-01

**Authors:** Lior Artzi, Bareket Dassa, Ilya Borovok, Melina Shamshoum, Raphael Lamed, Edward A Bayer

**Affiliations:** 1 Department of Biological Chemistry, The Weizmann Institute of Science, Rehovot, Israel; 2 Department of Molecular Microbiology and Biotechnology, Tel Aviv University, Ramat Aviv, Israel

**Keywords:** Cellulosomes, Cellulases, Cohesin, Dockerin, Scaffoldin, CBM, Glycoside hydrolases, Biomass degradation, Biofuels

## Abstract

**Background:**

*Clostridium clariflavum* is an anaerobic, thermophilic, Gram-positive bacterium, capable of growth on crystalline cellulose as a single carbon source. The genome of *C. clariflavum* has been sequenced to completion, and numerous cellulosomal genes were identified, including putative scaffoldin and enzyme subunits.

**Results:**

Bioinformatic analysis of the *C. clariflavum* genome revealed 49 cohesin modules distributed on 13 different scaffoldins and 79 dockerin-containing proteins, suggesting an abundance of putative cellulosome assemblies. The 13-scaffoldin system of *C. clariflavum* is highly reminiscent of the proposed cellulosome system of *Acetivibrio cellulolyticus*. Analysis of the *C. clariflavum* type I dockerin sequences indicated a very high level of conservation, wherein the putative recognition residues are remarkably similar to those of *A. cellulolyticus*. The numerous interactions among the cellulosomal components were elucidated using a standardized affinity ELISA-based fusion-protein system. The results revealed a rather simplistic recognition pattern of cohesin-dockerin interaction, whereby the type I and type II cohesins generally recognized the dockerins of the same type. The anticipated exception to this rule was the type I dockerin of the ScaB adaptor scaffoldin which bound selectively to the type I cohesins of ScaC and ScaJ.

**Conclusions:**

The findings reveal an intricate picture of predicted cellulosome assemblies in *C. clariflavum.* The network of cohesin-dockerin pairs provides a thermophilic alternative to those of *C. thermocellum* and a basis for subsequent utilization of the *C. clariflavum* cellulosomal system for biotechnological application.

## Background

In today’s world, the plant cell wall is one of industry's most common raw materials and provides the main component of fabric, paper, and wood. These materials, as well as byproducts from agriculture eventually end up as cellulosic waste and are a major source of pollution [1,2]. The plant cell wall contains a variety of polysaccharides, including cellulose as the major component. Cellulose is a crystalline polysaccharide, which is constructed from glucose monomers linked together by β-1,4-linkages [3-6]. An efficient way to degrade cellulose to single glucose molecules will lead to potential recycling of the cellulose and conversion of the glucose subunits to bioethanol and/or other useful chemicals by a simple fermentation step. Today, we rely on fossil fuels as a primary energy source, and the ability to harvest the energy encapsulated in biomass can help liberate society from the complete dependence on unsustainable fuel sources [7-9].

The cellulosome is a high-molecular-weight, multi-enzyme complex, found in anaerobic bacteria and has the ability to efficiently degrade cellulosic substrates [6,10-12]. Cellulosomes are secreted from the bacterial cell and may then be anchored to the cell surface or found in the free state in the extracellular medium. The cellulosome was first discovered in the anaerobic, thermophilic bacterium *Clostridium thermocellum*[13-15]. It is composed of two types of protein components: the structural proteins (scaffoldins) and the enzymatic subunits. The scaffoldins are non-catalytic proteins that carry cohesin modules, which are responsible for the integration of the enzyme subunits into the complex [10,12,16]. Scaffoldins that bind enzymes are called primary scaffoldins, and they usually contain type I cohesins [17-21]. Cellulolytic enzymes contain a type I dockerin module that interacts specifically with a type I cohesin module found on the scaffoldin. In this way, the enzymes can integrate into the scaffoldins and create the large multi-enzyme cellulosome complexes. Some of these primary scaffoldins contain a dockerin module, which gives them the ability to assemble with another scaffoldin called the adaptor scaffoldin, first seen in the bacterium *Acetivibrio cellulolyticus*[22]. This arrangement allows attachment of the cellulosome to the cell surface via successive interactions, first between the primary and adaptor scaffoldins, and then via a cell-anchoring scaffoldin. The primary scaffoldin may also interact directly with the anchoring scaffoldin that fastens the cellulosome to the cell surface via an S-layer homology (SLH) domain [23,24]. This architectural flexibility multiplies the possible enzyme compositions of the cellulosome [12]. In *A. cellulolyticus*, the adaptor scaffoldin bears type II cohesins that specifically interact with the type II dockerin of the primary scaffoldin. In addition, a scaffoldin may contain a carbohydrate-binding module (CBM) that allows the cellulosome to target specific carbohydrate substrates [20,25-28]. The scaffoldins and enzymes are the building blocks of the cellulosome, and the specific cohesin-dockerin interactions give rise to extensive assemblage possibilities and a variety of complexes.

*Clostridium clariflavum* is a gram-positive, anaerobic, thermophilic, spore-forming bacterium that was first discovered and isolated from an anaerobic sludge taken from a thermophilic methanogenic bioreactor. It has shown the ability to utilize cellulose and cellobiose, the only source of carbon and energy [29-31]. Studies of 16S rRNA-based phylogenetic have revealed that *C. clariflavum* and *C. thermocellum* are closely related. Interestingly, another closely related species to *C. clariflavum* is *A. cellulolyticus*, an anaerobic, mesophilic, cellulolytic bacterium, with a complicated cellulosomal system containing 16 scaffoldins and 143 putative dockerin-containing proteins [32-34]. These properties of *C. clariflavum* render it of prime interest for further exploration, and motivate us to reveal its cellulosome system and enzymes. Discovery of novel, potent cellulolytic enzymes and cellulosomes from these bacterial species may help the development of methods for efficient degradation of cellulose. The entire *C. clariflavum* genome was sequenced and putative enzymes (cellulosomal and non-cellulosomal) were revealed, such as bifunctional glycoside hydrolases with or without a dockerin module, and putative scaffoldins [31].

In the current study, we investigated the genes that include presumed cellulosomal modules (for example, cohesins, dockerins and CBMs). Using DNA sequence data, we were able to bioinformatically characterize dockerin-bearing proteins and cohesin-containing scaffoldins of *C. clariflavum*, and compare them with the cellulosomal proteins of *C. thermocellum* and *A. cellulolyticus*. Recombinant cohesin and dockerin modules that were identified from the *C. clariflavum* genome were cloned into matching fusion-protein cassettes and expressed. The modules served for evaluation of the various cohesin-dockerin interactions which then allowed us to predict potential cell-bound and cell-free cellulosomal complexes in this newly described cellulosome-producing bacterium.

## Results

### Variety of cohesin-containing proteins

**Figure 1:**
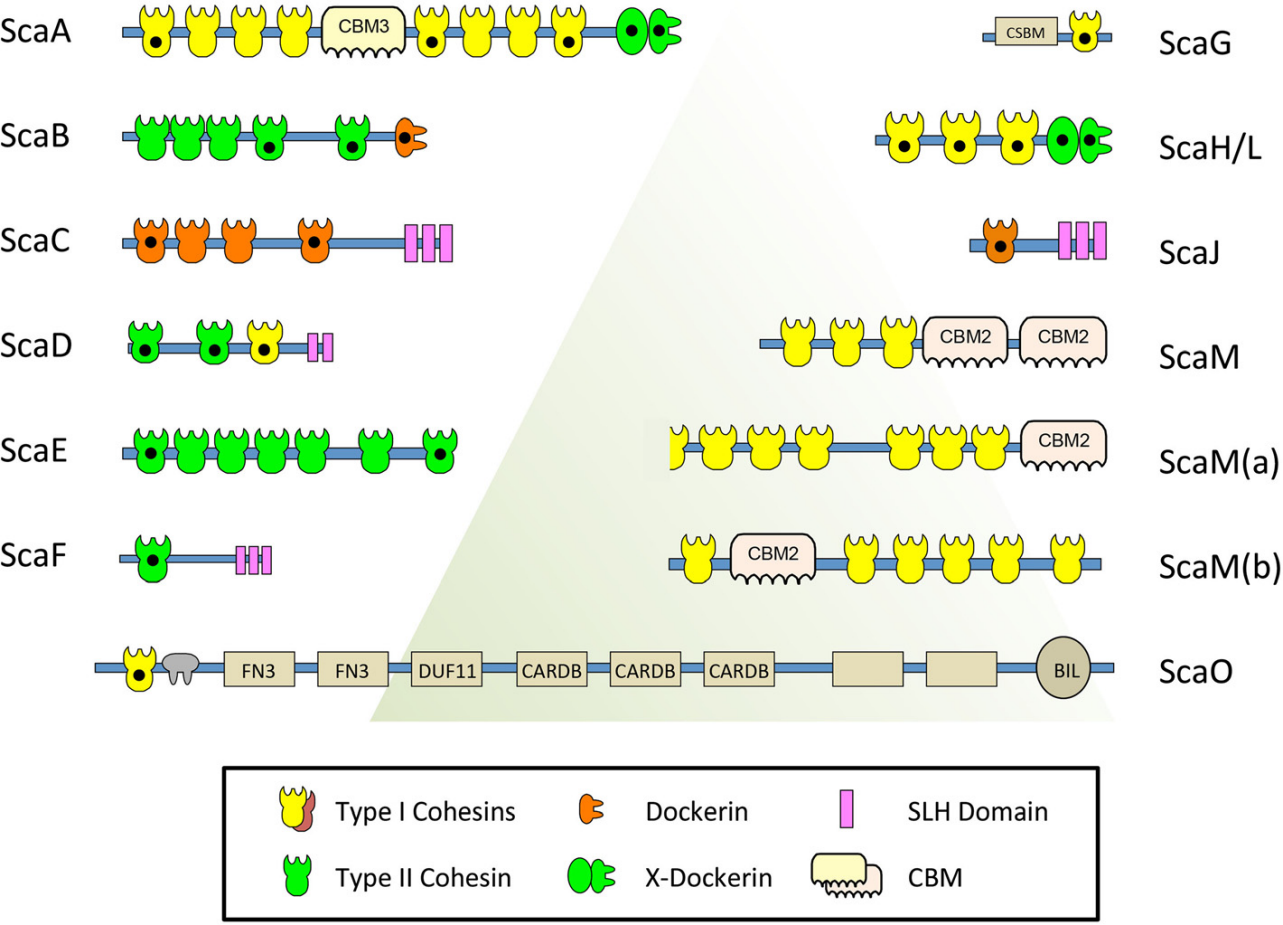
**Pictograms showing modular arrangement of putative scaffoldins of the *C. clariflavum *DSM 19732 genome.** Thirteen putative scaffoldins were identified bioinformatically. Black dots indicate cohesin and dockerin modules of the designated scaffoldins that were expressed and examined for specific interactions in the current study. All sequences contain an N-terminal signal peptide except ScaO and ScaM(a). CBM, carbohydrate-binding module; CSBM, cell surface-binding module; FN3, fibronectin type III domain; CARDB, cell adhesion-related domain found in bacteria; DUF11, domain of unknown function (Pfam PF01345); BIL, bacterial intein-like domain; SLH, S-layer homology. Accession numbers of *C. clariflavum* scaffoldins: [YP_005047733 (ScaA), YP_005047732 (ScaB), YP_005047731.1 (ScaC), YP_005047730 (ScaD), YP_005046332 (ScaE), YP_005047223 (ScaF), YP_005046504 (ScaG), YP_005047817 (ScaH/L), YP_005047757 (ScaJ), YP_005048513 (ScaM), YP_005048561 (ScaM(a)), YP_005048562 (ScaM(b)), YP_005046147 (ScaO): GeneBank].

**Figure 2:**
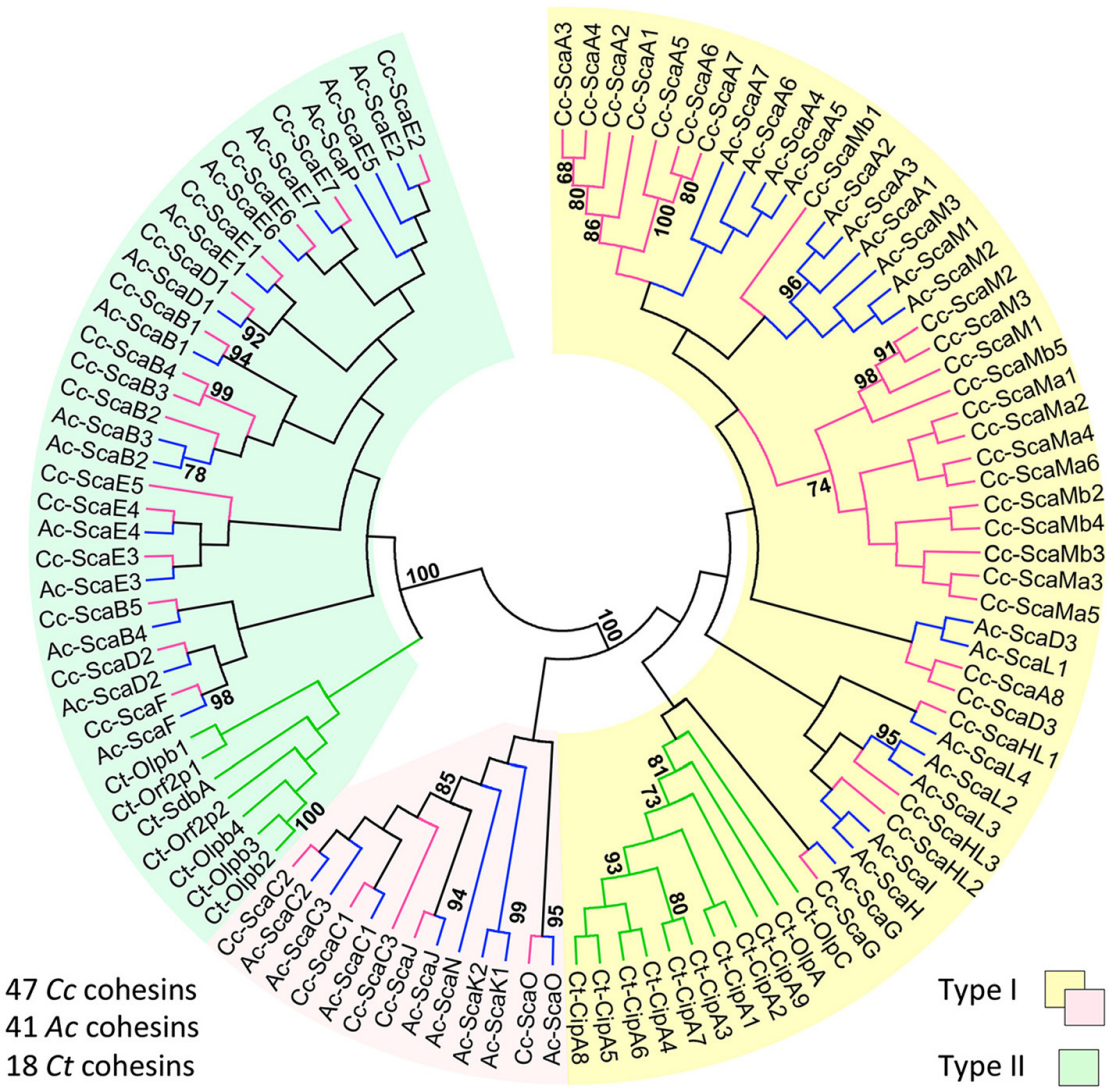
**Phylogeny of *C. clariflavum *cohesins.** A set of 47 *C. clariflavum* (Cc), 41 *A. cellulolyticus* (Ac), and 18 *C. thermocellum* (Ct) cohesin-like modules, derived from deduced amino-acid sequences (supporting Additional file 1: Figure S1), was aligned using the CLUSTALW2 program at the EBI website [35], which then served to reconstruct an unrooted phylogenetic tree by the MEGA5.10 software [36], using the neighbor-joining method with 500 bootstrap replicates. Numerical values above the nodes indicate bootstrap percentiles. The cohesin-like modules distribute into two major classes: type I (yellow) and type II (green). Among the type I cohesin-like modules one subgroup is separated from the majority of the modules (pink).

Recently, the 4.9 Mbp genome of *C. clariflavum* DSM 19732 was sequenced and annotated [31]. We further investigated the presumed cohesin-containing proteins and identified 49 cohesin modules distributed among 13 different scaffoldins (Figure 1), some of which carry both dockerin and cohesin modules on the same protein. Among these modules, two cohesins seem to be truncated. The other 47 complete putative cohesin sequences were aligned with cohesins from *A. cellulolyticus* and *C. thermocellum* (Additional file 1: Figure S1) and are presented on a phylogenetic tree (Figure 2), divided into type I and type II cohesins by sequence. Fifteen cohesin modules are classified as type II and 34 cohesin modules are classified as type I. The type I cohesin modules are separated into two groups, which may suggest the division of the type I group into subtypes. The putative *C. clariflavum* cohesin sequences are closely related to those of *A. cellulolyticus* on the phylogenetic tree. For the majority of the cohesin modules of *A. cellulolyticus* there is a homolog in the *C. clariflavum* genome. In contrast, the cohesin modules of *C. thermocellum* are clustered on a separate branch of the tree and are in general more distantly related to the *A. cellulolyticus* and *C. clariflavum* modules.

### Architecture and modular arrangement of the scaffoldins

The modular organization of the cohesin and dockerin modules on the 13 different scaffoldins is represented in Figure 1. The names of the scaffoldins are based on the homology of the cohesin modules of *C. clariflavum* to the cohesin modules from *A. cellulolyticus*, according to Dassa *et al*. [34]. The scaffoldins possess a signal peptide at their N-terminus (except ScaO and ScaM(a)), suggesting that the scaffoldins are secreted from the cell. Most of the scaffoldins carry only one type of cohesin (type I or type II), except ScaD which contains both types of cohesins: the first two ScaD cohesins are type II, and the third is type I. This unique type of scaffoldin is very similar to the ScaD scaffoldin of *A. cellulolyticus* [ZP_09464030] [31,37]. In addition, ScaD contains two repeats of SLH domains, which enable its anchoring to the cell wall.

Likewise the homology between other scaffoldins of *C. clariflavum* and *A. cellulolyticus* is high, and the modular organization of the proteins is very similar. For example, the primary scaffoldin ScaA is similar to ScaA [ZP_09464033.1] of *A. cellulolyticus*[21] and to CipA [CAA47840] of *C. thermocellum*. Eight type I cohesins are located in its sequence, like the ScaA of *A. cellulolyticus* (7 type I cohesins) and CipA (9 type I cohesins) of *C. thermocellum*, and all contain a CBM3 module (Dassa *et al*. 2012 [34]). At the C-terminus of the three scaffoldins there is an X-dockerin (XDoc) modular dyad, which was shown previously to bind type II cohesins [22,38-41]. In fact the *C. clariflavum* ScaA parallels closely ScaA of *A. cellulolyticus,* both in its overall modular architecture (Figure 1 and Dassa *et al*. [34]) and in the sequences of its various cohesin modules (Figure 2); the major difference being the unique presence of an N-terminal catalytic module (family 9 cellulase) in the *A. cellulolyticus* scaffoldin, which is lacking in *C. clariflavum* ScaA, and instead contains an extra cohesin in the same position.

Most of the scaffoldins from *C. clariflavum* have a homologous scaffoldin in *A. cellulolyticus*. Notably, the adaptor scaffoldin ScaB and cell-anchoring scaffoldin ScaC have equivalent proteins in *A. cellulolyticus* [ZP_09464032 (ScaB) and ZP_09464031 (ScaC)]. ScaE consists exclusively of seven type II cohesins, which are closely related to the seven cohesin modules of ScaE from *A. cellulolyticus* [ZP_09465494] and Cthe_0736 from *C. thermocellum*.

ScaG has a single type I cohesin and a region annotated as a copper-amine-oxidase-like domain. Intriguingly, both *A. cellulolyticus* and *C. thermocellum* genomes include scaffoldins that are composed of the same modular type, that is, ScaG [ZP_09464788] and OlpC [YP_001036883], respectively. Interestingly, Pinheiro *et al*. [42] have demonstrated that the ‘copper-amine-oxidase-like domainʼ of *C. thermocellum* OlpC is responsible for binding to the secondary cell wall polymers that are bound to the S-layer in gram-positive bacteria, thereby allowing the anchoring of OlpC to the cell wall of *C. thermocellum.* From the sequence similarity of this domain in OlpC and ScaG, it therefore seems likely that this domain in ScaG would exhibit the same cell-surface anchoring function, and the domain is thus designated cell surface-binding module (CSBM).

In addition to ScaG, there are two additional scaffoldins that are composed of a single cohesin module and a cell-anchoring module. In this context, ScaF consists of a type II cohesin and three SLH domain repeats. ScaJ, however, contains a type I cohesin and also three SLH domain repeats. ScaH/L has three type I cohesins which are related phylogenetically to the cohesins on ScaH [ZP_09462752] and ScaL [ZP_09464968] of *A. cellulolyticus* (Dassa *et al*. [34], Figure 2).

*C. clariflavum* possesses three scaffoldins with CBM2 modules and type I cohesins (ScaM, ScaM(a), ScaM(b)). Previously, only ScaM from *A. cellulolyticus* [ZP_09463433] was reported to be a unique scaffoldin that bears CBM2 modules [34]. All other previously described scaffoldins contain CBM modules from family 3. Family-2 CBMs are usually attached to enzymes and bind to various polysaccharides such as cellulose and xylan [43]. In this case, ScaM of *C. clariflavum* is similar to ScaM of *A. cellulolyticus* [ZP_09463433] with its three type I cohesins and two CBM2 modules. Moreover, the cohesins of the three scaffoldins ScaM, ScaM(a) and ScaM(b) are phylogenetically related to the *A. cellulolyticus* ScaM cohesins (Figure 2).

The pair of ORFs, ScaM(a) and ScaM(b) was found in a unique arrangement on the *C. clariflavum* genome. The ORF of Clocl_4212 (ScaM(b), [YP_005048562] codes for a protein with a signal peptide, at least six cohesin modules and a CBM2 module. The ORF seems to be truncated, because it ends with an N-terminal half of a cohesin, while the second ORF, Clocl_4211 (ScaM(a), [YP_005048561] starts with a complementary C-terminal half of the cohesin (and no signal peptide), having at least six cohesins and a C-terminal CBM2 module. Both ORFs overlap on the genome, suggesting that they may reflect a single extended ORF, which underwent a frame shift. In addition, the nature of these ORFs is remarkably repetitive due to the close similarity (near-identity) of the cohesin modules, which did not allow us to validate the transcript of this locus by PCR.

Finally, a unique scaffoldin, ScaO, bears a type I cohesin and a type I dockerin at the N-terminus of the protein. This protein does not contain a signal peptide, which suggests it is not secreted from the bacterium, but does not rule out the possibility for secretion. ScaO has two putative fibronectin type III domains, three cell adhesion-related domains, and a bacterial intein-like domain. The rest of the protein includes unknown domains. The designation of this protein is in accordance to the architectural similarity of its N-terminal portion with portions of ScaO from *A. cellulolyticus.*

### Dockerin-containing proteins

A large set of genes encoding 79 dockerin-containing proteins is present in the *C. clariflavum* genome; 75 of them have type I dockerins whereas four possess type II dockerins, which, similar to *C. thermocellum* and *A. cellulolyticus*, have an X-module upstream of the dockerin module. The 75 type I dockerin-containing proteins have a variety of predicted catalytic units that are distributed on 48 dockerin-containing enzymes. These enzymes include 41 glycoside hydrolases (GHs) from 15 different families, 14 carbohydrate esterases (CEs) and 2 polysaccharide lyases (PLs), whereby some of the dockerin-containing enzymes contain more than one catalytic module and are thus bifunctional [31]. Some of the dockerin-containing proteins can be classified as non-catalytic components, for example, the serpin- (Clocl_3968) and expansin-containing proteins (Clocl_1298 and Clocl_1862). In others, CBMs are the only identifiable modular type, and many others contain modules of unknown function; hence these latter dockerin-containing proteins cannot currently be classified as enzymes. Multiple sequence alignment of the dockerins and the annotated modules located in each parent protein can be found in Additional file 2: Figure S2.

**Figure 3:**
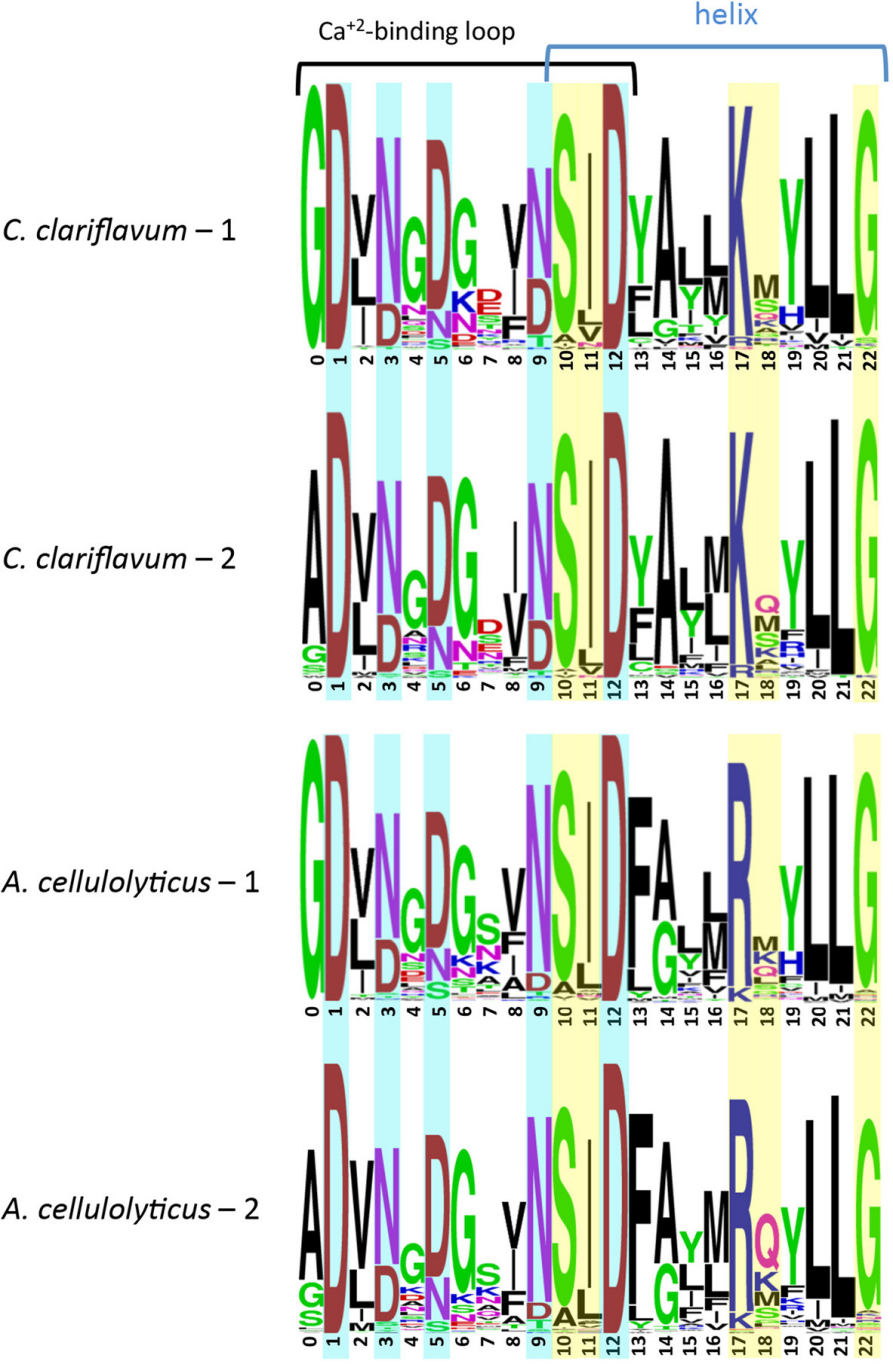
**Comparative sequence logos of the *C. clariflavum *and *A. cellulolyticus *dockerin modules.** Amino acid conservation of the type I dockerin repeat sequences was performed by a logo, created using WebLogo (see Methods) based on 74 type I dockerin sequences of *C. clariflavum* and 138 of *A. cellulolyticus*. The top logo of each represents the first dockerin sequence repeat and the bottom logo represents the second dockerin repeat. Calcium-binding residues are highlighted in light blue, and the presumed recognition residues responsible for cohesin-dockerin interactions are highlighted in yellow.

Sequence conservation of the dockerin was demonstrated by performing multiple sequence alignment of the 75 type I dockerins, and creating a sequence logo of the two repeats of the dockerin modules (Figure 3). Most of the dockerin-containing proteins have two repeats of the duplicated sequence, whereby each repeat contains a predicted Ca^+2^-binding loop and an alpha-helix, with a linker separating the two repeats. Only one protein, Clocl_2271, has a single repeat of dockerin module, located at the N-terminus of the protein. Notably, the Ca^+2^-binding repeats are highly conserved in both repeats and the coordinating residues are located at positions 1, 3, 5, 9 and 12. The predicted residues critical for cohesin-dockerin recognition are residues 10, 11, 17, 18, and 22 [44-46]. Most of the latter residues are highly conserved in *C. clariflavum*, except residue 18 which is variable. Interestingly, the predicted recognition residues of the *C. clariflavum* dockerins are highly similar to those of *A. cellulolyticus.* Three of the dominant residues are identical (S, I and G in positions 10, 11 and 22) and a fourth very similar (K versus R in position 17 of *C. clariflavum* and *A. cellulolyticus,* respectively).

**Figure 4:**
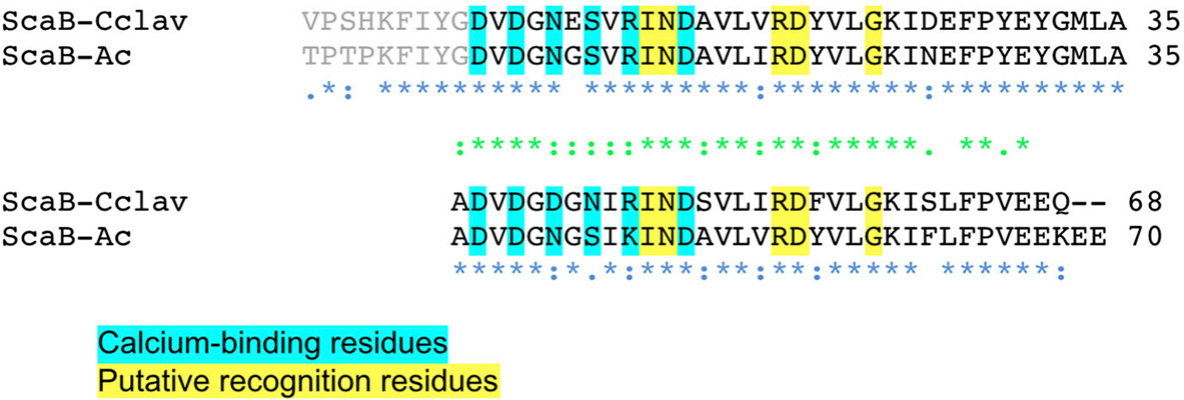
**Sequences of *C. clariflavum *and *A. cellulolyticus *ScaB dockerins*.*** Sequence alignment of the two dockerin modules was performed using the CLUSTALW2 program at the EBI website. Consensus residues are as defined accordingly; *indicates a position which has an identical residue, and colon (:) and period (.) indicate conservation between groups of strongly and weakly similar properties, respectively; blue indicates conservation between species and green indicates conservation between the two repeated segments. Ca^+2^-binding residues are highlighted in cyan, and putative recognition residues are highlighted in yellow. Residues are numbered relative to the highly conserved glycine (designated 0), which is positioned adjacent to the initial calcium-binding aspartate (residue 1).

Significantly, the predicted recognition residues of the type I ScaB dockerin is different from all other type I dockerins in *C. clariflavum.* Notably, its sequence is remarkably similar to that of the ScaB dockerin of *A. cellulolyticus* (Figure 4). Moreover, the predicted recognition residues (I, N, R, D, G of the designated positions) are identical between the two sequence repeats and between the two species.

### Selection and design of cohesin- and dockerin-modules for interaction studies

The multiplicity and complexity of the cellulosomal components of *C. clariflavum* enables diverse architectural assemblies of the cellulosome. In order to identify and characterize the relevant interactions among the cellulosomal components, we employed the matching fusion-protein system and affinity-based ELISA approach, developed previously in our laboratory [47]. For this purpose, we chose representative cohesin and dockerin modules and expressed them in two different cassettes: each cohesin module was N-terminally fused to a CBM3a module originating from the CipA scaffoldin of *C. thermocellum*[26]. Within the context of the present work, this type of chimera is termed CBM-Coh. The counterpart - the dockerin module - was fused to the C terminus of xylanase T6 from *Geobacillus stearothermophilus*[48], and this type of chimera is herein termed XynDoc for the type I dockerins and Xyn-XDoc for the type II dockerins. This fusion protein system was originally developed with the purpose of achieving high-level protein expression, and for increasing the stability and solubility of the cohesin and dockerin modules. Both the thermostable xylanase T6 and CBM3a have indeed been shown to elevate expression levels in *Escherichia coli* cells and assist in protein solubility. The CBM3a module also allows efficient purification via its cellulose affinity properties [26]. Following protein expression, SDS-PAGE analysis of the purified proteins revealed single protein bands in agreement with their calculated molecular mass (data not shown).The cohesins that were selected for expression are shown in Figure 1 and are labeled with a black dot. Nineteen cohesins were expressed in order to detect interactions with various dockerins. The cohesins that were expressed are as follows (enumerated from the N to the C-terminus of the given protein): cohesins 1, 5 and 8 from ScaA; cohesins 4 and 5 from ScaB; cohesins 1 and 4 from ScaC; cohesins 1 and 7 of ScaE; the single cohesins of ScaF, ScaG, ScaJ and ScaO; and all three cohesins of ScaD and ScaH/L (Figure 1).

Four dockerin modules were selected for expression. Three of these dockerins were from the scaffoldins: two type II dockerins were taken from ScaA and ScaH/L, along with their N-terminal X modules. A type I dockerin was taken from ScaB, and another dockerin was taken from the GH48 enzyme [YP_005048367.1] of *C. clariflavum*. The three dockerins from the scaffoldin proteins were chosen in order to identify interactions between the different scaffoldins and to determine how the cellulosome assembles. The dockerin of the GH48 enzyme was selected as generally representative of the cellulosomal enzymes that are believed to bind type I cohesins (Figure 3). The GH48 enzyme of *C. clariflavum* exhibits high sequence similarity to the Cel48S enzyme from *C. thermocellum*[31] that was found to be the most abundant enzyme in the *C. thermocellum* cellulosome [14,49-52]. It seems likely that due to its abundance, the GH48 dockerin would interact with the vast repertoire of type I *C. clariflavum* cohesins. In this context, the putative recognition residues are consistent with the dominant residues shown in Figure 3.

### Characterization of cohesin-dockerin interactions

**Figure 5:**
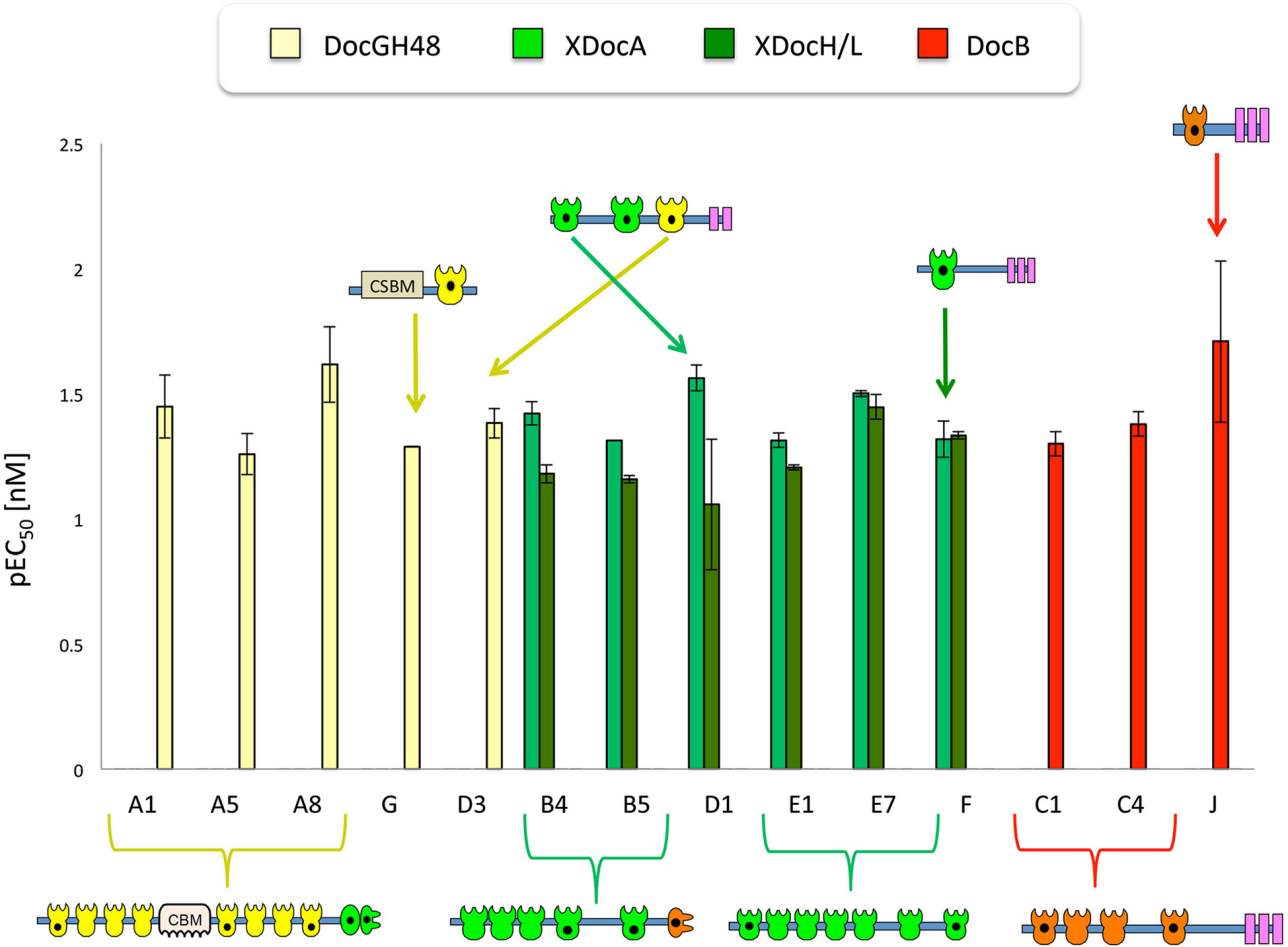
**Determination of cohesin-dockerin specificity by affinity-based ELISA.** In order to identify specific interactions between the nineteen CBM-Cohs and the four XynDocs, microtiter plates were coated with the respective CBM-Coh fusion protein, and increasing concentrations of the various XynDocs were then applied to the plates. The EC_50_ was calculated for the resultant interactions, and values of the pEC_50_ are presented on the y-axis in the bar graph. Coh, cohesin; Doc, dockerin; XDoc, X-dockerin modular dyad; CBM, carbohydrate-binding module. The cohesin names and numbers are shown on the horizontal axis (for example, A1 indicates the first cohesin of ScaA). Xyn-XDocA, green; Xyn-XDocH/L, dark green; XynDocB, red; XynDocGH48, yellow.

All three ScaA cohesins examined in this work exhibited significant interaction with the XynDocGH48 (Figure 5), whereas the other XynDocs that were tested did not bind the ScaA cohesins significantly (that is, below the detection threshold shown in Figure 5). The type I cohesin, CohG, interacted with the XynDocGH48 similarly to the ScaA CBM-Cohs.

The ScaB cohesins were classified bioinformatically as type II cohesins and were thus expected to interact with type II dockerins. The ELISA results verified this anticipated result, and the strongest interaction was detected between CBM-Cohs B4 and B5 and Xyn-XDocA, which also displayed a strong interaction with the Xyn-XDocH/L. The interactions detected for the type II cohesins of ScaE (cohesins E1 and E7) and ScaF further conform to this rule. It thus appears that the type II cohesins generally interact with the type II dockerins in this organism.

Like the ScaA cohesins, the cohesins of ScaC were classified as type I cohesins. Nevertheless, CBM-Cohs C1 and C4 failed to interact with the type I XynDocGH48 but did interact with the type I XynDocB. These results correspond with the findings described by Xu *et al*. 2003 [22], for ScaB [ZP_09464032] and ScaC [ZP_09464031] from *A. cellulolyticus*, and anticipated by the status of the putative recognition residues. In this context, the residues of the ScaB dockerin (Figure 4) are very different from those of the enzyme-borne dockerins (Figure 3). As mentioned above, the recognition sequences of the ScaB dockerins from both species are identical, and it is thus not surprising that interspecies cross-reactivity was observed between representative ScaC cohesins and the ScaB dockerins (data not shown).

ScaJ was also classified as type I cohesin, and, like the ScaC cohesins, its CBM-Coh interacted exclusively with XynDocB. This specificity pattern for the ScaB dockerin reflects the phylogenetic status of the cohesins. The cohesin modules of ScaC and ScaJ from both *C. clariflavum* and *A. cellulolyticus* are located on a separate branch of the type I cohesins in the phylogenetic tree (Figure 2). These findings, together with the pattern of interactions that these modules display, suggest that ScaC and ScaJ belong to a subtype of cohesin modules that is distinct from the rest of the type I modules.

The unique scaffoldin ScaD, which according to its gene sequence bears 2 type II cohesins (cohesins D1 and D2) and one type I cohesin (cohesin D3), was shown to bind two different types of dockerins. CBM-CohD1 showed the strongest interaction with Xyn-XDocA and a moderately high interaction with Xyn-XDocH/L. However, CBM-CohD3 exhibited type-specific binding to XynDocGH48. These results are compatible with the work of Xu *et al*. 2004 [37], which have shown the same pattern of binding for ScaD [ZP_09464030] from *A. cellulolyticus*. The existence of two different types of cohesins renders ScaD as both a primary scaffoldin (binds enzymes) and anchoring scaffoldin (binding other scaffoldins to the cell surface). The presence of this singular type of scaffoldin in both *C. clariflavum* and *A. cellulolyticus* appears to be a defining feature of these two cellulosome-producing species.

Five CBM-Cohs (H/L1, H/L2, H/L3, D2 and O) that were examined showed no significant binding to either of the XynDocs, using the affinity-based ELISA approach. Although CBM-Cohs H/L1, H/L2, H/L3 and O do not show strong interactions with any of the dockerins, they had a preferential but very weak binding to XynDocGH48 (below the threshold shown in the graph). Moreover, CBM-CohD2 also failed to show detectable interactions with any of the XynDocs. In order to examine whether this was related to the concentration of the reactants, we increased the coating concentration of the CBM-Cohs at 25 nM of CBM-CohD2, which then promoted a significant interaction between CBM-CohD2 and Xyn-XDocA and Xyn-XDocH/L, thus suggesting a weaker but specific cohesin-dockerin interaction in these cases. There remains the possibility that the CBM-CohD2 might be relatively unstable and subject to misfolding or denaturation, which would also account for the observed results.

## Discussion

As fossil fuel reserves are exhausted, the industrialized world will require large supplies of renewable energy resources in order to maintain the current quality of life without consuming the natural energy supplies on earth. Recycling of dedicated biofuel crops and biomass waste will become an essential and primary part of the solution for future energy demands. Many cellulolytic bacteria capable of degrading polysaccharide substrates have been discovered and explored [6,53-58], and research into new cellulolytic species will enrich our knowledge of ecofriendly biomass degradation. Owing to the purported efficient cellulolytic properties of cellulosomes, the characterization of the relatively limited number of bacteria that produce them is of special interest. Progress in the field will thus pave the way for industrialized use of cellulolytic enzymes in green energy production.

The exploration of the cellulosomal genes of *C. clariflavum* has revealed a modular protein construction set that allows the assembly of intricate multi-enzyme architectures. This structural and enzymatic complexity is likely a key to the bacterium's reported highly efficient cellulose-degradation capabilities [29]. We investigated the putative cohesin- and dockerin-containing genes and identified 13 scaffoldins, which contain a variety of modules and domains that are distributed among the 13 polypeptide chains. *C. clariflavum* and *A. cellulolyticus* show high sequence homology [31] and display similar scaffoldin architectures and high homology among their cohesin modules (Figure 2). Similarly, the type I dockerin sequences of *C. clariflavum* are closely related to *A. cellulolyticus* dockerins and exhibit similar cohesin recognition residues. In contrast to this remarkable similarity, the glycoside hydrolases of *C. clariflavum* show high homology to those of *C. thermocellum* rather than *A. cellulolyticus*[31]. As can be seen from these findings, *C. clariflavum* appears to have acquired characteristics from both *C. thermocellum* and *A. cellulolyticus*. In this context, it is a thermophilic bacterium like *C. thermocellum* and has an exceptionally complex set of cellulosomal components like *A. cellulolyticus*[29,31,34].

**Figure 6:**
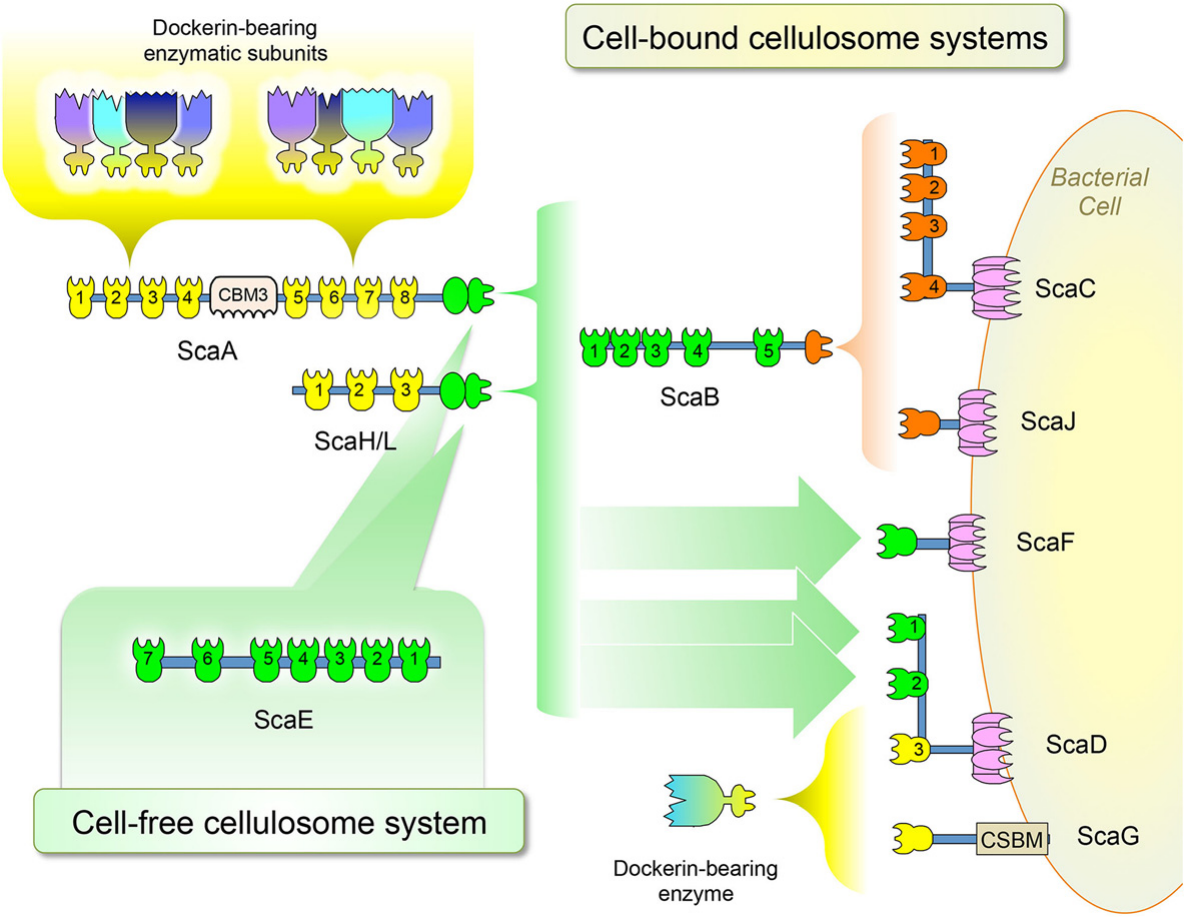
**Proposed architectures for cell-bound and cell-free cellulosome assembly in *C. clariflavum*.** The scheme shows the possible interactions among scaffoldin and enzymatic modules*,* as derived from examination of interactions by affinity ELISA. Specification of scaffoldins is detailed in Figure 1. Four potential cell-anchored cellulosomal complexes are represented. Two of the complexes employ the adaptor protein ScaB to join the cell-anchored scaffoldins (ScaC and ScaJ, containing an SLH domain, and four and one type I cohesins, respectively) to the primary enzyme-integrating scaffoldins (ScaA and ScaH/L) via the type II cohesins of ScaB and XDocs of the former. The type II cohesins of ScaD (cohesins 1 and 2) and ScaF are also cell-anchored scaffoldins that bind directly ScaA or ScaH/L. The type I cohesins of ScaG and ScaD (cohesin 3) interact with type I dockerins of dockerin-bearing enzymes. ScaG is suspected to be a cell-anchored scaffoldin, based on previous studies of the copper amine-oxidase domain in the OlpC protein from *C. thermocellum*. ScaE has seven type II cohesins which are able to bind seven XDoc modules, thereby creating a large, cell-free cellulosomal complex. CBSM, cell surface-binding module.

The detected cohesin-dockerin interactions from the affinity-ELISA studies suggest a large number of possible cellulosome architectures. Figure 6 shows the many possible complexes that can be formed. Based on the interactions among the cellulosomal components of this study, one can appreciate the complexity of such complexes by examining the interconnectivities possible for ScaC. ScaC has three SLH domains, which would together serve to anchor the complex to the cell surface, and four cohesins that have the ability to bind four ScaB dockerins. ScaB, in turn, bears five cohesins and can thus bind the XDoc modular dyad of ScaA. ScaA can then bind multiple type I dockerins, borne by the various *C. clariflavum* enzymes. Such a complex, with fully occupied cohesins, would thus theoretically include 160 enzymatic units, thus rendering the multi-enzyme *C. clariflavum* system the largest cellulosomal complex yet discovered, superseding the deduced architectures of *C. thermocellum* (63 enzymes), *A. cellulolytics* (96 enzymes) and *B. cellulosolvens* (110 enzymes). Similarly, a cellulosome constructed of ScaJ as the anchoring scaffoldin would create a complex with 40 enzymatic units, a cellulosome built with ScaD as its anchoring scaffoldin would represent a complex of 17 enzymes, and a cellulosome based on ScaF would result in a complex of 8 enzymes - all having ScaA as the primary scaffoldin. The remarkable diversity of these cell-bound cellulosome assemblies in *C. clariflavum* mirrors that of the *A. cellulolyticus* assemblies, as they have a homologous set of anchoring scaffoldins. This diversity appears to reflect the elaborate surface morphology observed previously for *A. cellulolyticus.*

The ScaE-based cellulosomal complex appears to be the only potential cell-free cellulosome system in this bacterium that would catalyze plant cell wall polysaccharides independent of the location of the bacterial cell and may thus enhance the decomposition of recalcitrant substrates into simpler oligosaccharide units. ScaE is composed of seven type II cohesins and has no dockerin module, SLH domain, CBM module, or other detectable sequence that would anchor it to the cell surface or to the polysaccharide substrate. However, its seven type II cohesins are capable of binding the XDoc modules of ScaA. A cellulosome constructed of ScaE and 7 ScaAs would contain 56 enzymes and would supply 7 CBM3 modules, which can direct the complex to substrate. This type of scaffoldin is also produced in both *A. cellulolyticus* and *C. thermocellum,* that is, ScaE [34] and Cthe_0736 [16], respectively - both of which also contain seven type II cohesins. The presence of this type of scaffoldin in these three bacteria emphasizes its apparent importance in complex cellulosome systems. In contrast, the simple cellulosome systems of mesophilic clostridia, such as *C. cellulolyticum, C. cellulovorans* and *C. papyrosolvens,* all contain a single primary scaffoldin alone and lack scaffoldins that bear type II cohesins [12,59].

It is important to note that all the type II cohesins examined in the current study have shown interaction with the XDoc of both ScaA and ScaH/L. Combined interaction with the two scaffoldins allows for a large number of possible cellulosome assemblies. Nevertheless, no significant interaction was detected between the ScaH/L cohesins and the tested dockerins, although these cohesins may interact with other dockerin-containing enzymes which were not tested in this work. In order to expand our knowledge of the specificity of dockerin-containing enzymes incorporated into the cellulosome, more dockerin modules from *C. clariflavum* genome will be investigated in the future.

## Conclusions

In this work we revealed a novel, complicated, intriguing cellulosomal system that has the potential to help us understand the cellulosomal conversion of recalcitrant polysaccharide substrates to simple sugars and their subsequent conversion to biofuels. The multiplicity of cellulosomal components in *C. clariflavum* and their possible interactions and interconnectivities observed gives rise to the formation of diverse complexes that enable efficient cellulose degradation. Furthermore, the stability of *C. clariflavum* proteins is expected to be higher in comparison to mesophilic bacteria such as *A. cellulolyticus*, as *C. clariflavum* is a thermophilic bacterium [29-31]. Until the discovery of a cellulosome system in *C. clariflavum,* the only thermophile known to produce a cellulosome system has been *C. thermocellum.* Our findings suggest that *C. clariflavum* can be further developed into a good source for new potent cellulose-degrading enzymes and novel cellulosomal architectures, thus providing a thermophilic cellulosome-producing alternative to the prototypical *C. thermocellum* system.

## Materials and methods

### Genomes source

Genome sequences of *C. clariflavum* DSM 19732 [CP003065], *A. cellulolyticus* CD2 [NZ AEDB02000000], and *C. thermocellum* ATCC 27405 [CP000568] were obtained from the GenBank of NCBI [60].

### Sequence analysis and database searches

BLASTP algorithm [61] searches were performed for predicted proteins of *C. clariflavum*, using deduced amino acid sequences of the known cohesin and dockerin modules as queries. Hits above an E-value of 10^-4^ were examined individually, by searching for characteristic sequence features. For example, for dockerin modules, we searched for two Ca^+2^-binding repeats, putative helices and linker regions.

Multiple sequence alignments (MSAs) were created using the CLUSTALW servers, at the EBI [35] and at the PBIL [62]. When needed, MSAs generated by the EBI CLUSTALW2 server were used to reconstruct phylogenetic trees in the MEGA 5.10 software [36] using the neighbor-joining method with 500 bootstrap replicates. Amino acid sequence logos were performed using the WebLogo application, version 2.8.2 [63].

### Annotation of dockerin-containing enzymes

In order to identify and analyze enzymatic modules of the dockerin-containing proteins of *C. clariflavum* DSM 19732, the proteins were annotated using the Carbohydrate Active Enzymes database (CAZY) [64,65]. The analysis was based on sequence conservation between catalytic modules, and the different catalytic modules were sorted into different family types, such as GHs, glycosyltransferases, PLs, CEs and CBMs.

### Source of *C. clariflavum* genomic DNA

*C. clariflavum* DSM 19732 was supplied by the Leibniz Institute DSMZ-German Collection of Microorganisms and Cell Cultures. The bacterium was grown on GS-2 medium. Genomic DNA was extracted by Dr Harish Kumar Reddy (Tel Aviv University), as described earlier [66].

### Cloning and design of CBM3a-Cohesin plasmid cassettes

The pET28a plasmid was used to create fusion proteins CBM3a-Cohesin (CBM-Coh). The PCR product of the CBM3a module from *C. thermocellum* scaffoldin CipA [26] was inserted into the pET28a plasmid by using NcoI and BamHI sites as previously described by Barak *et al*. 2005 [47]. Genes encoding cohesin modules were cloned using specific primers (Additional file 3: Table S1) by PCR from *C. clariflavum* genomic DNA using Reddymix *x*2 (Advanced Biotechnologies Ltd., Epsom, Surrey, United Kingdom). The amplified DNA fragments were purified by The HiYield gel-PCR fragment extraction kit (Real Biotech Corporation, RBC, Banqiao City, Taiwan). Cohesin inserts were restricted by BamHI (5' terminus) and XhoI (3' terminus) FastDigest enzymes (Thermo scientific, Fermentas UAB, Vilnius, Lithuania) and ligated into the pET28a-CBM3a cassette [40,47]. The plasmids were transformed into an *E. coli* XL-1 Blue strain and purified via QIAprep spin miniprep kit (QIAGEN GmbH, D-40724 Hilden, Germany).

### Xylanase-dockerin (Xyn-Doc) cassettes

A PCR product of *G. stearothermophilus* T6 xylanase with a His-tag and BspHI (5' terminus) and KpnI (3' terminus) restriction sites was obtained [40,47,48,67] and inserted into the pET9d vector. The dockerin modules were produced using specific primers (supplementary material) by PCR with the KpnI site at the 5' terminus and the BamHI at the 3' terminus. The dockerin-encoding genes were inserted into the plasmid using KpnI and BamHI enzymes.

### Protein expression

The pET28a cassette containing the CBM-Coh fusion proteins and the pET9d cassette containing the XynDoc fusion proteins were transformed into *E. coli* BL21 (DE3) strains and plated onto LB-kanamycin plates. For each plate, 4 to 5 mL of Luria-Bertani broth (LB) were added in order to resuspend the cells. The cells were added to 1L of LB with 50 μg/mL kanamycin (Sigma-Aldrich, Rehovot, Israel) and 2 mM CaCl_2_ and were grown for 2.5 h at 37°C to A_600_ ≈ 0.8 to 1.0. Induction for protein expression was made by adding Isopropyl-1-thio-β-D-galactoside (IPTG) (Fermentas UAB, Vilnius, Lithuania) in a final concentration of 0.2 mM, and the growth was continued in 16°C for 16 h. Cells were harvested by centrifugation at 5,000 rpm for 15 minutes.

### CBM-Coh purification

After centrifugation, cells were resuspended with 30 mL TBS (Tris-buffered saline, 137 mM NaCl, 2.7 mM KCL, 25 mM Tris-HCl, pH = 7.4), and protease-inhibitor cocktail was added (1 mM PMSF, 0.4 mM benzamidine and 0.06 mM benzamide). The cells were sonicated and the supernatant was centrifuged for 30 minutes at 15,000 rpm at 4°C. The supernatant was then added to 2 g of macroporous bead cellulose preswollen gel (IONTOSORB, usti nad Labem, Czech Republic) and incubated for 1 h, with rotation at 4°C. The mixture was then loaded onto a gravity column and washed with 100 mL of TBS that contained 1 M NaCl, and then washed with 100 mL TBS. Three 10-mL elutions of 1% triethanolamine (TEA) were then collected. The three fractions were subjected to SDS-PAGE in order to assess protein purity, and then dialyzed with TBS containing 5 mM CaCl_2_.

### Xyn-Doc purification

After centrifugation, cells were resuspended with 30 mL TBS supplemented with 5 mM imidazole and protease-inhibitor cocktail. Cells were disrupted by sonication and centrifuged for 30 minutes at 15,000 rpm at 4°C. The purification was performed in a batch purification system as described previously by Vazana *et al*. 2010. Fractions of 2 mL were collected, and protein purity was assessed by SDS-PAGE. The fractions that contained the protein were pooled and dialyzed with TBS and 5 mM CaCl_2_.

### Protein concentration and storage

Proteins concentrations were evaluated by absorbance at 280 nm, based on the extinction coefficients derived from the known composition of amino acids of each protein. Extinction coefficients were calculated using the ExPASy ProtParam tool [68]. The proteins were concentrated by Amicon ultra concentrators (Millipore, Carrigtwohill, Co. Cork, Ireland), and stored at -20°C in 50% (vol/vol) glycerol.

Affinity-based ELISA was performed by the protocol reported earlier by Barak *et al*. 2005 [47]. The 96-well ELISA plates (Nunc, A/S, Roskilde, Denmark) were coated with the fusion proteins CBM-Cohs at a concentration of 3 nM, and variable concentrations of Xyn-Docs (ranging between 2 pM and 20 nM) were used to detect specific cohesin-dockerin interactions. The interactions with the four XynDocs proteins were examined immunochemically by using anti-xylanase primary antibody and horseradish peroxidase (HRP)-labeled secondary antibody. For comparative purposes, pEC_50_ was calculated for each binding curve as described earlier [47,69] and the results were presented in bar graph form.

## Abbreviations

CBM: carbohydrate-binding module; CE: carbohydrate esterase; Coh: cohesin; CSBM: cell surface-binding module; Doc: dockerin; ELISA: enzyme-linked immunosorbent assay; GH: glycoside hydrolase; MSA: multiple sequence alignment; ORF: open reading frame; PL: polysaccharide lyase; SLH: S-layer homology; XDoc: X module coupled with a type II dockerin; Xyn: xylanase.

## Competing interests

The authors declare that they have no competing interests.

## Authors’ contribution

LA and EAB designed the research. LA and MS performed the experiments. LA, RL and EAB analyzed the results. BD and IB analyzed the genome data. LA, BD and EAB wrote the manuscript. All authors read and approved the manuscript.

## Supplementary Material

Additional file 1: Figure S1 Multiple sequence alignment of 106 cohesin sequences originated from the genomes of *C. clariflavum* (Cc), *A. cellulolyticus* (Ac) and *C. thermocellum* (Ct). Alignment length: 148; identity (*): 1 residue = 0.67%; strongly similar (:): 3 residues = 2.03%; weakly similar (.): 3 residues = 2.03%; different: 141 residues = 95.27%. All the accession numbers for *C. clariflavum* cohesin-containing proteins can be found in Figure 1, and the accession numbers of *A. cellulolyticus* and *C. thermocellum* cohesin-containing proteins can be found in Dassa *et al*. 2012 [34]. Click here for file

Additional file 2: Figure S2 Multiple sequence alignment of the *C. clariflavum* 74 dockerin modules. Cyan highlight indicates putative calcium-binding residues. Yellow highlight indicates putative recognition residues. Gray highlight marks the last C-terminal residue of a corresponding protein. x indicates a computational fusion of Clocl_2272 [YP_005046783] and Clocl_2271 [YP_005046782] to reconstruct a complete dockerin motif (a stop codon TAA of Clocl_2272 was replaced with NNN). BIL, bacterial intein-like domain; CARDB, cell adhesion-related domain found in bacteria; CBM, carbohydrate binding module (followed by family number); CE, carbohydrate esterase (followed by family number); COH, cohesin; DOC, dockerin; EXPN, expansin; FN3, fibronectin type III domain; GH, glycoside hydrolase (followed by family number); LNK, linker; PL, polysaccharide lyase (followed by family number); Serpin, serine protease inhibitor; SIGN, signal peptide; UNK, unknown region; X, X domain. Alignment length: 84. Identity (*): 5 identical residues = 5.62%. Strongly similar (:): 1 residue = 1.12%. Weakly similar (.): 3 residues = 3.37%. Different: 80 residues = 89.89%. Click here for file

Additional file 3: Table S1 List of primers for the *C. clariflavum* cohesin and dockerin modules that were cloned in this study. Nucleotides shown in bold indicate restriction sites added to the primers. Click here for file
