# Supplementary material for: Cellulosomics of the cellulolytic thermophile Clostridium clariflavum
Source: Biotechnol Biofuels. 2014 Jul 1;7:100. doi: 10.1186/1754-6834-7-100 (PMC4582956; doi:10.1186/1754-6834-7-100)
Supplement: Additional file 1: Figure S1 — Multiple sequence alignment of 106 cohesin sequences originated from the genomes of C. clariflavum (Cc), A. cellulolyticus (Ac) and C. thermocellum (Ct). Alignment length: 148; identity (*): 1 residue = 0.67%; strongly similar (:): 3 residues = 2.03%; weakly similar (.): 3 residues = 2.03%; different: 141 residues = 95.27%. All the accession numbers for C. clariflavum cohesin-containing proteins can be found in Figure 1, and the accession numbers of A. cellulolyticus and C. thermocellum cohesin-containing proteins can be found in Dassa et al. 2012 [34]. [file 1754-6834-7-100-S1.pdf]

Cohesin name	Alignment	
Ac-ScaC1	GKVSGEPG--SIVSVPVTFSN------VPAT---GIYALSFSVNFDKSMLSVVSVEPGSL	49
Cc-ScaC1	GRVSGEPG--SIVTVPVTFSN------VPAT---GIYALSLNLNFDSTKISVVSVEPGSL	49
Ac-ScaC3	GSTSGKAG--SVVSVPITFTN------VPKS---GIYALSFRTNFDPQKVTVASIDAGSL	49
Ac-ScaC2	GSVSGKPG--SVVSVPITFSN------VPKS---GIYALSFRIGYDNTKIRVVGVESGSL	49
Cc-ScaC2	GSISGKPG--DTVNVPIKFYN------VPAS---GIYTLSFRVGYDKNKLSVVGVEPGAL	49
Cc-ScaC3	GSVNAEKGKDSQVEVPVYFEN------VPEV---GIYVCSLFIKFDETKLKVVEVKPQEL	51
Ac-ScaJ	AAVNGDPG--TTVTVPLTLKN------VPSS---GIYTCSFWVTYDPQTFTSVSIASGNL	49
Cc-ScaJ	AVLRGEKG--TTVTVPLTVKN------VPES---GIYTCSFWVDFNPEIFSSVSITPGEL	49
Ac-ScaK1	SSVYGRNG--DIVIIPIQFQKG----EVPSE---GISTFGFSLSYDSNVFDLVSVNKTID	51
Ac-ScaK2	GSGSGQKG--DVVKIPVRFLKEPPNLEVPSN---GISYFGFVLRYDSNLFEEASFLQTDI	55
Ct-CipA4	DTVNAKPG--DTVRIPVRFSG------IPSK---GIANCDFVYSYDPNVLEIIEIEPGDI	49
Ct-CipA5	DTVNAKPG--DTVRIPVRFSG------IPSK---GIANCDFVYSYDPNVLEIIEIEPGDI	49
Ct-CipA6	DTVNAKPG--DTVRIPVRFSG------IPSK---GIANCDFVYSYDPNVLEIIEIEPGDI	49
Ct-CipA8	DTVNAKPG--DTVRIPVRFSG------IPSK---GIANCDFVYSYDPNVLEIIEIEPGDI	49
Ct-CipA3	DTVNAKPG--DTVNIPVRFSG------IPSK---GIANCDFVYSYDPNVLEIIEIKPGEL	49
Ct-CipA7	DTVNAKPG--DTVRIPVRFSG------IPSK---GIANCDFVYSYDPNVLEIIEIEPGEL	49
Ct-CipA2	GKVTGSVG--TTVEIPVYFRG------VPSK---GIANCDFVFRYDPNVLEIIGIDPGDI	49
Ct-CipA9	GRAEGRPG--DTVEIPVNLYG------VPQK---GIASGDFVVSYDPNVLEIIEIEPGEL	49
Ct-CipA1	GKVTAAVG--SKVEIPITLKG------VPSK---GMANCDFVLGYDPNVLEVTEVKPGSI	49
Ct-OlpA	GNVKARPG--DRIEVPVSLKN------VPDK---GIVSSDFVIEYDSKLFKVIELKAGDI	49
Ct-OlpC	EGVKAKAG--DTVEIPLKFEN------VPSH---GIQSFNLSLYYDSKAIEVLKVEPGSI	49
Ac-ScaA2	DSVNGNVG--EQIVVPVSFAN------VPSN---GISTADMTITYDSSKLEYVSGDAGSI	49
Ac-ScaA3	DSVNGNVG--EQIVIPVSFAN------IPAN---GISTADMTITYDSSKLEYVSGVPGSI	49
Ac-ScaA1	DSVNGNVG--EQIVVPVSFAN------VPSN---GVSTADMTITYDSSKLEYVSGAAGSI	49
Ac-ScaM1	GSASGENG--ATIVVPVNFAN------VPSN---GISTADMTITYDSSKLEYVKGEAGSI	49
Ac-ScaM2	DSVSGNNG--STIVVPVSFAN------IPSS---GISTADMTIMYDSSKLEYVSGEAGSI	49
Ac-ScaM3	DSVSGGYG--TTIEVPIRFAN------VPSS---GISTADMTITYDSSKLEYVSGAAGSI	49
Cc-ScaM2	ESVTANAG--QQVVVPIKLAN------VPSN---GITTADMTIIYDPSKLEYVNGSAGSI	49
Cc-ScaM3	ESVTANAG--QQVVVPIKLAN------VPSN---GITTADMTIIYDPSKLEYVNGSAGSI	49
Cc-ScaM1	ESVTANVG--QQVVVPIKLAN------VPSN---GITTADMTITYDPSKLEYVNGSAGSI	49
Cc-ScaMa1	ESVTASAG--EQVVVPIKLAN------VPSN---GITTADMTITYDASKLEYVKGEAGSI	49
Cc-ScaMa2	ESVTASAG--EQVVVPIKLAN------VPSN---GITTADMTITYDASKLEYVKGEAGSI	49
Cc-ScaMa3	ESVTASAG--EQVVVPIKLAN------VPSN---GITTADMTITYDASKLEYVKGEAGSI	49
Cc-ScaMa4	ESVTASAG--EQVVVPIKLAN------VPSN---GITTADMTITYDASKLEYVKGEAGSI	49
Cc-ScaMa5	ESVTASAG--EQVVVPIKLAN------VPSN---GITTADMTITYDASKLEYVKGEAGSI	49
Cc-ScaMa6	ESVTASAG--EQVVVPIKLAN------VPSN---GITTADMTITYDASKLEYVKGEAGSI	49
Cc-ScaMb2	ESVTASAG--EQVVVPIKLAN------VPSN---GITTADMTITYDASKLEYVKGEAGSI	49
Cc-ScaMb3	ESVTASAG--EQVVVPIKLAN------VPSN---GITTADMTITYDASKLEYVKGEAGSI	49
Cc-ScaMb4	ESVTASAG--EQVVVPIKLAN------VPSN---GITTADMTITYDASKLEYVKGEAGSI	49
Cc-ScaMb5	ESVTASAG--GQVVVPIKLAN------VPSN---GITTADMTITYDASKLEYVKGEAGSI	49
Cc-ScaMb1	ESVSGSFG--QQIVVPVKFSN------VPSN---GIITADMTITYDSSKLEYVSGEAGSI	49
Ac-ScaA4	GAVQGGVG--ETVTVPVTMTK------VPTT---GVSTADFTVTYDATKLEYVSGAAGSI	49
Ac-ScaA5	ANVSGNAG--SEVVVPVSIEG------VSAN---GVSAADFTITYDATKLDYVSGAAGSI	49
Ac-ScaA6	DTVEGNPG--SSVVVPVKLSG------ISKN---GISTADFTVTYDATKLEYISGDAGSI	49
Ac-ScaA7	DTVKAATG--TQVVVPVSFVN------VPAT---GISTTDMTITYDATKLQYVSGDAGSI	49
Ac-ScaD3	GSYVANYG--DEVTVPVKYVN------VPAK---GISTTDMTIIYDKNMLKYLSGEVGSI	49
Cc-ScaA3	GSVEGKSG--ETITVPINFIK------VPSN---GVTTCDLTITYDATKLEYVSGEAGSI	49
Cc-ScaA4	GSVEGKSG--ETITVPVNFIK------VPSN---GVTTCDLTITYDATKLEYVSGEAGSI	49
Cc-ScaA2	GSVEGKSG--ETITVPINFIK------VPSN---GITTCDLTITYDATKLEYVSGGAGSI	49
Cc-ScaA1	ESVEGKSG--ETITVPVKFSN------VSSVSGAGITTCDLTITYDATKLEYVSGEAGSI	52
Cc-ScaA6	GKVEGKAG--ETVTVPVTLTN------VSAN---GVTSADFAITYDSSVLEYVSYEAGSI	49
Cc-ScaA7	GKVEGKAG--ETVTVPVTLTN------VSAN---GVTSADFAITYDSSVLEYVSYEAGSI	49
Cc-ScaA5	GKVEGKAG--ETVTVPVTLTK------VPAN---GITSADFAITYDSSVLEYVSYEAGSI	49
Cc-ScaA8	DTVNAAKG--QEVIVPVQLIN------VPEG---GLAAADMTIIYDATKLEYVDIEAGSI	49
Cc-ScaD3	DSDYAAYD--QEIIIPVRLRN------VPSR---GITTADITINYDAKKLKFIEVTAGNI	49
Ac-ScaL2	GTLNAAEN--DNITLPINYMN------VPAN---GISACNFTITYDPQQLEYISYEPGNI	49
Ac-ScaL3	GSKQASPN--ENVTLPIYFDD------VPAI---GISTCDFTITYDPKQLEFLSYEPGSI	49
Ac-ScaL1	GSVNSQTG--KEVCVPIKLTC------VPDN---GIVSTDMSISYDTSILKYTGGSAGDI	49
Cc-ScaHL1	ASEHKELD--GNVIIPITFSN------VPEK---GILTCDMTIVYDPTKLEYISCDIGDI	49
Ac-ScaL4	GSGDAKIG--DTITIPVNFEN------VPDK---GIAACNMTITYDSTQLEFVENEVGSI	49
Ac-ScaG	ATVEAKAG--DVVEIPITFSN------ISKI---GIATIDFTVTYNAANLEFIEAPAGDI	49
Cc-ScaHL2	GSANGNTG--ELVTVPINFLN------VPEN---GISTVDIEVTYNPTLLEFVSAAPGAV	49
Ac-ScaI	GSIEGKVG--DLVTIPISFVN------VSPY---EIETVDMIITYDPTQLEYVSGEAGPL	49
Ac-ScaH	GSSDAKYK--DQVNIPITFK-------GSPE---RISTLKMTITYDPTQLEYVSVSPGEI	48

```
Cc-ScaHL3       GSVNGYAN--DLVTVPIYFTS------IPFN---YIVSFDLTLNYDPSQLEYISYEPGEI 49
Ac-ScaD1        NKTSIELD--DIITATIKISN------IQKF-----AGYQVNIKFDPEVLQAVNPISGKE 47
Cc-ScaD1        NKTTVEVN--DIITATIKINN------IPKF-----SGLQVNIKFDPEVLQAVDPISGKP 47
Ac-ScaB1        DKTTASVG--EIVTASINIKN------ITNF-----SGCQLNMKYDPAVLQPVT-SSGVA 46
Cc-ScaB1        DKNKASVG--EIVTASINIKN------ITDF-----SGCHVNLKYDPNVLQPVT-SNGVA 46
Ac-ScaE3        DKTTASVG--DIINATVRVNG------ISNF-----AGYQVNLTYDPTVLKPVN-ALGQD 46
Cc-ScaE3        DNTEVEVG--DIINASIKVDN------IADL-----AGYQINLSYDPTVLKPVT-IDGKD 46
Ac-ScaE6        DKTTAKVN--DTIKATVRISN------IDNF-----SGYQASLKYDPTVLQPIT-SSGTA 46
Ac-ScaE7        DKTTAAVG--ETITATVRVNK------ISNF-----SGYQLNLKYDPAVLQPIK-SSGAS 46
Cc-ScaE6        DKTCAKVN--DTITATIMVEN------IDNL-----AGYQVNLKYDPNMLQPVT-SSNTP 46
Cc-ScaE7        DKTSAKVG--EIIKAIIKVNE------IKNL-----SGYQINLKYDPDVLQPVK-PSGAS 46
Ac-ScaE4        DKTTASVG--DIISATLKVEG------MADL-----AGYQVNLKYDPAVLQPVNPATGVA 47
Cc-ScaE4        DKTEASFG--DIITATLRADG------IDNL-----AGFQVNIKYDPKVLQPVNPATREP 47
Ac-ScaE5        DKTSAAVG--DTVKATLSINN------IDNF-----AGYQVNLNYDPEVLQPVT-ASGVA 46
Cc-ScaE5        DKNNPSVG--DIINASILILN------IDKL-----AGYQFNIKYDPEVLQPVD-SNGVP 46
Ac-ScaE1        DKTSAKVG--ETITATLNINN------INNF-----AGYQVNLKYDPAVLQPVK-STGAQ 46
Cc-ScaE1        DKTNAEIG--DIITATVYINN------IRNF-----AGYQVNLKYDPEVLQPVT-KAGKP 46
Ac-ScaF         DKTSAAVG--EIIKASVIVDN------VSGF-----AGYQVNIKYDPTVLEAVNPDTGAA 47
Cc-ScaF         DKTSAAVG--DIIKASIVVNN------VKGF-----AGYQVNIKYDPTVLEAINLDTGAA 47
Ac-ScaB2        DKNAAEVG--EIIKATVKINK------ITNF-----SGYQVNIKYDPTVLQAVNPKTGVA 47
Ac-ScaB3        DKNTAEVG--QIIKATVKINK------ITNF-----SGYQVNIKYDPTVLQAVNPKTGVA 47
Cc-ScaB2        DKNSAEVG--EIIKAVVKINN------IKNF-----SGYQVNIKYDPEVLEAVNPDTGEV 47
Cc-ScaB3        DKNSAEAG--EIITATLKVNK------ISNL-----SGYQVNIKYDPEVLQAVNPNTGRA 47
Cc-ScaB4        DKNSAEVG--EIITATLKVNK------ISNL-----SGYQVNIKYDPEVLQAVNPNTGRA 47
Ac-ScaB4        DKTDVSVG--DIIKASVRIND------ISNF-----SGYQVNIKYDTTVLQAVNPKTGAE 47
Cc-ScaB5        DKTEVSVG--DVIIASVRVND------IKNL-----SGYQVNIKYDTDVLKAVNPITKEA 47
Ac-ScaD2        DKTSVVVG--DIIKASIQINN------INNF-----AGYQVNIKYDPTILQAVNFNTKSP 47
Cc-ScaD2        NKDMVVVG--DIIKATISIKD------IDNF-----AGYQINIEYDPNILVPVDPNTGNP 47
Ac-ScaE2        DKTSAAVG--DIVKATLSVNN------IENF-----AGYQVNLKYDPAVLQPVT-ASGTP 46
Cc-ScaE2        DKTTAKVG--ETVKVYLSVNN------IFGL-----SGYQVNLKYDPDVLQPVT-SSGKP 46
Ac-ScaP         DKTYAEVG--DIVTATLRVNQ------ITNL-----SAYQVNIKYDPQVIQPVD-DDLQP 46
Ct-Olpb2        DKTKVKVG--DIITATIKIEN------MKNF-----AGYQLNIKYDPTMLEAIELETGSA 47
Ct-Olpb3        DKTKVKVG--DIITATIKIEN------MKNF-----AGYQLNIKYDPTMLEAIELETGSA 47
Ct-Olpb4        DKTKVKEG--DVIIATIRVNN------IKNL-----AGYQIGIKYDPKVLEAFNIETGDP 47
Ct-Orf2p1       DKTKANIG--DIIIATIRIDN------INNF-----SGYQLNIKYDPSYLQAVNPLTGEP 47
Ct-Orf2p2       DKTKVKVG--DVIVATVKAKN------MTSM-----AGIQVNIKYDPEVLQAIDPATGKP 47
Ct-Olpb1        DKTEVHVG--DVITATIKVNN------IRKL-----AGYQLNIKFDPEVLQPVDPATGEE 47
Ct-SdbA         DRNKGEVG--DILIGTVRINN------IKNF-----AGFQVNIVYDPKVLMAVDPETGKE 47
Ac-ScaO         PYLRANAG--DEIVVPVYIDS------VPQG---GICSFDFKVKYDKSVMSYTGYEAGAL 49
Cc-ScaO         PFIKVDSD--SFIEVPLMLDN------IPEE---GISCFNFAINYNKNILTYVGNSKGTL 49
Cc-ScaG         GTIKAEPA--TTIDVPINVSFD------TTR---GLNTFNLTLAFDNDNLEFVEVKPGDI 49
Ac-ScaN         ENKTIIAG--DTFTMDVMLSN------VPTG---GLAYGTVSVGFDKSKFTCTSIEKGPI 49
                            .   :                           ::   .

Ac-ScaC1        IEDPSDFNHYYN-----------------NV------NGLASMSFEAPVDGSRMIDNNGV 86
Cc-ScaC1        VEDPDDFALFTN-----------------NE------HGFTSMSFMAPADRSRIVDKDGV 86
Ac-ScaC3        IENASDFTTYYN-----------------NE------NGFASMTFEAPVDRARIIDSDGV 86
Ac-ScaC2        VEKAEDFNMFIN-----------------NT------YNFTSMTFEAPIDGSRMIKSDGV 86
Cc-ScaC2        IEDPRDMNYYTM-----------------TS------SGLTTITFEAPADETRKIRNDGV 86
Cc-ScaC3        IEDKSDFASHFD-----------------NTNINRFNSGFVSMSFMGPADDSRRINKDGV 94
Ac-ScaJ         ITNTNDFHSNVN-----------------SE------MGFVAMTFEAPADGSRMIKSDGT 86
Cc-ScaJ         IINPSDFYSNVN-----------------NT------KGFVAMVFEAPADESRVIKNDGT 86
Ac-ScaK1        VFYNNKN------------------------------NGYINFLCDKIIDADFIEKNDGV 81
Ac-ScaK2        VVANSDE------------------------------SGYYSFIGTKQINKNFIE-DGGV 84
Ct-CipA4        IVDPNPDKSFDT-----------------AVYPDRKI--IVFLFAEDSGTGAYAITKDGV 90
Ct-CipA5        IVDPNPDKSFDT-----------------AVYPDRKI--IVFLFAEDSGTGAYAITKDGV 90
Ct-CipA6        IVDPNPDKSFDT-----------------AVYPDRKI--IVFLFAEDSGTGAYAITKDGV 90
Ct-CipA8        IVDPNPDKSFDT-----------------AVYPDRKI--IVFLFAEDSGTGAYAITKDGV 90
Ct-CipA3        IVDPNPDKSFDT-----------------AVYPDRKI--IVFLFAEDSGTGAYAITKDGV 90
Ct-CipA7        IVDPNPTKSFDT-----------------AVYPDRKM--IVFLFAEDSGTGAYAITEDGV 90
Ct-CipA2        IVDPNPTKSFDT-----------------AIYPDRKI--IVFLFAEDSGTGAYAITKDGV 90
Ct-CipA9        IVDPNPTKSFDT-----------------AVYPDRKM--IVFLFAEDSGTGAYAITEDGV 90
Ct-CipA1        IKDPDPSKSFDS-----------------AIYPDRKM--IVFLFAEDSGRGTYAITQDGV 90
Ct-OlpA         VE--NPSESFSY-----------------NVVEKDEI--IAVLYLEETGLGIEAIRTDGV 88
Ct-OlpC         IT--DPANNFDY-----------------NIVYKDSE--IVFLFDDDKQKGEGLIKTDGV 88
Ac-ScaA2        VT--NPTVNFGI-----------------NKETDGK---LKVLFLDYTMS-TGYISTNGV 86
Ac-ScaA3        VT--NPDVNFGI-----------------NKETDGK---LKVLFLDYTMS-TGYISTSGV 86
```

```
Ac-ScaA1      VT--NPTVNFGI-----------------NKEADGK---LKVLFLDYTMS-TGYISTNGV 86
Ac-ScaM1      VT--NASTNFGI-----------------NKESDGK---IKALFLDYTMS-NGYISQSGV 86
Ac-ScaM2      VT--NASTNFGI-----------------NKESDGK---VKALFLDYTMS-NGYISQSGV 86
Ac-ScaM3      VT--NAATNFAI-----------------NKEAVGK---IKVLFLDYTMS-REYISNNGV 86
Cc-ScaM2      VM--NPSVNFAI-----------------NKEDNGI---IKILFLDETLL-NEYIREDGI 86
Cc-ScaM3      IM--NPSVNFAI-----------------HKENNGT---IKILFLDETLL-NEYIREDGI 86
Cc-ScaM1      VM--NPSVNFAI-----------------NKENNGT---IKILFLDETLL-NEYIREDGI 86
Cc-ScaMa1     VI--NPSINFAI-----------------NKEKDGI---IRILFLDDTLL-NEYISKDGV 86
Cc-ScaMa2     VI--NPSINFAI-----------------NKEKDGI---IRILFLDDTLL-NEYISKDGV 86
Cc-ScaMa3     VI--NPSINFAI-----------------NKEKDGI---IRILFLDDTLL-NEYISKDGV 86
Cc-ScaMa4     VI--NPSINFAI-----------------NKEKDGI---IRILFLDDTLL-NEYISKDGV 86
Cc-ScaMa5     VI--NPSINFAI-----------------NKEKDGI---IRILFLDDTLL-NEYISKDGV 86
Cc-ScaMa6     VI--NPSINFAI-----------------NKEKDGI---IRILFLDDTLL-NEYISKDGV 86
Cc-ScaMb2     VI--NPSINFAI-----------------NKEKDGI---IRILFLDDTLL-NEYISKDGV 86
Cc-ScaMb3     VI--NPSINFAI-----------------NKEKDGI---IRILFLDDTLL-NEYISKDGV 86
Cc-ScaMb4     VI--NPSINFAI-----------------NKEKDGI---IRILFLDDTLL-NEYISKDGV 86
Cc-ScaMb5     VI--NPSINFAI-----------------NKEKDGI---IRILFLDDTLL-NEYISKDGV 86
Cc-ScaMb1     VT--NPSINYAI-----------------NKDKDGS---IKVLFLDDTLT-SGYISRDGV 86
Ac-ScaA4      VT--NPDVNFGI-----------------NKEADGK---IKVLFLDYTMA-TEYISKDGV 86
Ac-ScaA5      VK--NPDVNFGI-----------------NKEADGK---LKVLFLDYTMA-TEYISADGI 86
Ac-ScaA6      VT--NPGVNFGI-----------------NKESDGK---LKVLFLDYTMS-TGYISTDGV 86
Ac-ScaA7      VT--NPGVNFGI-----------------NKEADGK---LKVLFLDYTMT-TQYISEDGV 86
Ac-ScaD3      VS--NPNTNFGI-----------------NKEADGK---LKVLFLDYTMS-DGYISSDGV 86
Cc-ScaA3      VT--NPSVNFAI-----------------NKEDDGI---IKLLFLDETLV-SQYISEDGV 86
Cc-ScaA4      VT--NPSVNFAI-----------------NKEDDGI---IKLLFLDETLV-SQYISEDGV 86
Cc-ScaA2      VT--NPSVNFSL-----------------NKEEDGK---IKILFLDETLV-SQYISEDGV 86
Cc-ScaA1      VT--NPRVNFAL-----------------NKEEDGK---IKILFLDETLV-SQYISEDGV 89
Cc-ScaA6      VK--NPSVNLAL-----------------HKEKDGL---LNVLFLDETLI-SEYIAEDGV 86
Cc-ScaA7      VK--NPSVNLAL-----------------HKEKDGL---LNVLFLDETLI-SEYIAEDGI 86
Cc-ScaA5      VK--NPSVNLAL-----------------NKEKDGL---LKVLFLDETLI-NEYIAEDGI 86
Cc-ScaA8      VI--NPRINFAA-----------------HKEADGI---VKILFLDESFV-DQYISEDGD 86
Cc-ScaD3      IT--NPKINFAA-----------------HKEKDGV---IKLLFLDDTLR-YEYIRTDGI 86
Ac-ScaL2      VP--NPDVNFAI-----------------NKAEDGC---LKLLFLDHTIG-NECINFDGI 86
Ac-ScaL3      VP--NPDTNFSL-----------------VKTSEGS---LKLLFIDFTME-NEFIKSGGI 86
Ac-ScaL1      VP--DPDANFAI-----------------AETSPGV---LKLLFVDTTNL-DKHICTDGV 86
Cc-ScaHL1     VI--NPETNLVV-----------------SKVYDGL---IKLLFLDETLG-NELITSDGI 86
Ac-ScaL4      VK--NPDMNFAI-----------------NKLEDGT---IKILFVDCTMK-DEYITSNGL 86
Ac-ScaG       VK--NAGVNYGS-----------------NKSEDGS---IKFLFLDYTMTGAESINSNGV 87
Cc-ScaHL2     IP--SPDQNFNY-----------------NTSSESS---INIQYSKDSET-PACITSNGE 86
Ac-ScaI       IK--EAKLEYGV-----------------NKKSDGK---IEIFTFD-PFMGECYINSDGV 86
Ac-ScaH       IP--SPVSSFGT-----------------QLKSPGE---IELFFMNYTCCTDYDIRAEGM 86
Cc-ScaHL3     LK--ESDFCFGI-----------------EKISDGK---LNLSYLN---YIEGYIYKSGL 84
Ac-ScaD1      YQ--SSTMPLPG-DLLNNSD-FLSFSKAVNNLDNGELNICKSYITLEAYKNSGTEESTGS 103
Cc-ScaD1      YN--NSTLPLAG-ELLNNSE-FISFAQATNDLKNGIINISKSYLNLEAYREKGIDENTGS 103
Ac-ScaB1      YT--KSTMPGAG-TILN-SD-FNLRQVADNDLEKGILNFSKAYVSLDDYRTAAAPEQTGT 101
Cc-ScaB1      YK--NSTTPESG-TILKNTD-YGINLVASNKVEEGILNFSRSYLNLKSYREDANPESTGT 102
Ac-ScaE3      YT--NSTIPTSG-TLLGNYE-YGPYPVAAHNLSGGILNFGKSYTNLEAYRASGSAETSGT 102
Cc-ScaE3      YK--NTTLPLKG-DIIVNND-YSPYFIASHNLSAGTLNFTGSYLDLEAYRNSGNAETKGT 102
Ac-ScaE6      YT--NSSIPLSG-TILTNGD-YGTIPAASHNITAGKLNFGKLYTNLEDYRASGIPEETGT 102
Ac-ScaE7      YT--NSSIPAAG-TLIANSD-YGPVIAVSHNLTSGLLNFGKLYTNIEEYRASGSPEETGT 102
Cc-ScaE6      YT--NSSIPSKG-TLISNAN-FGPISAASHKLSEGILNFGRLYTNLADYRASQAPEKTGS 102
Cc-ScaE7      YT--NGTIPSSG-TLISNEE-FYPLNAVSHDLSKGILNFGKFYILLDDYKNSGTIEDTGT 102
Ac-ScaE4      YA--KSTNPTAG-DLLNNSD-YGVLPMASHDLEAGILNFSRSYTSLEDYRTNGN-EGPGK 102
Cc-ScaE4      YN--SNTNPEEG-DIIVNSE-FEPVSFALHDLKSGILNFSRSYTNLEAYRASES-EKSGI 102
Ac-ScaE5      YT--NNTIPTTG-EMLSNQD-YGILPIAYHDLTNGVLNFSKSYTNLEDYRLSGSPEKTGT 102
Cc-ScaE5      YT--NETIPAAG-GLINNED-FGIFAVASHNLEKGILNFSKLYTDLEGYKKSGKPETLGI 102
Ac-ScaE1      YT--NSTVPESG-TLLKNSD-FGVLPQASNNITSGILNFGKVYTNLEDYKANGKAESNGS 102
Cc-ScaE1      YT--NSTNPESG-TLLNNSE-FGAFAQALNNIEEGILNFGKVYTRLEDYRSSAEAESSGS 102
Ac-ScaF       FT--NSTNPKNG-TILNDAT-YGALPIASNNILQGILNFGKTYTSLEGYKASGSAETSGT 103
Cc-ScaF       FT--NTTVPQSG-TILNNSE-YGVLPIASHNVSQGILNFSKAYTNLNSYKASGSAETTGT 103
Ac-ScaB2      YT--NSSLPTSG-ELLVSED-YGPIVQGVHKISEGILNLSRSYTALEVYRASESPEETGT 103
Ac-ScaB3      YT--NSSLPTSG-ELLVNED-YGPIVQGVHKISEGILNLSRSYTALDVYRASESPEETGT 103
Cc-ScaB2      YT--NSTVPTSG-DLLKSEE-YGPFSKAENKVTEGILNFGSLYTNLSAYRADGRPEETGT 103
Cc-ScaB3      YT--NSTIPTSG-ELFTSED-YGVLPNAAHNIEKGILNFSRSYINLKAYREDGVAEETGT 103
Cc-ScaB4      YT--NSTIPTSG-ELFTSED-YGVLPNAAHNIEKGILNFSRSYINLKAYREDGVAEETGT 103
Ac-ScaB4      YK--NDSLPLEG-EILANQD-YSPFPTCDNIISEGLLNFGKLYTALDVYKESNLAEKSGV 103
Cc-ScaB5      YT--NNSLPLDG-EILINDE-YSIFSKAESNVNEGILNFSKGYVNLKEYRESNKPEESGS 103
```

```
Ac-ScaD2        FK--ISTLPLDG-SILLNSN-YSPFAYADNHVDKGILNFSKAYLGLEGYRTGGVAETSGV 103
Cc-ScaD2        YR--NYTFPTNG-NILQNNK-YIPLEKVNHDLEKGILNFGKTYLNLEEYRKSGKAESSGT 103
Ac-ScaE2        FS--KSTVPSGA-TILTNED-YYKYPMASHNISEGILCFSNTYTNLEDYKASGIAETTGT 102
Cc-ScaE2        YS--SSTNLSGG-DLIKNSD-YSPFYQASHNLEEGILNFAASYLNLPEYRASNNAETTGT 102
Ac-ScaP         YC--EDTVPTGA-TILNNSK-YSPMSIASNNLYSGILNFGAGYMNLLSYKQSGIAQTSGI 102
Ct-Olpb2        IA--KRTWPVTGGTVLQSDN-YGKTTAVANDVGAGIINFAEAYSNLTKYRETGVAEETGI 104
Ct-Olpb3        IA--KRTWPVTGGTVLQSDN-YGKTTAVANDVGAGIINFAEAYSNLTKYRETGVAEETGI 104
Ct-Olpb4        ID--EGTWPAVGGTILKNRD-YLPTGVAINNVSKGILNFAAYYVYFDDYREEGKSEDTGI 104
Ct-Orf2p1       IK--KRTMPAVNGTVLLKGDQYSITEVVENNVDEGILNFGKGYANLTEYRKSGKPETTGI 105
Ct-Orf2p2       FT--KETLLVDP-ELLSNRE-YNPLLTAVNDINSGIINYASCYVYWDSYRESGVSESTGI 103
Ct-Olpb1        FT--DKSMPVNR-VLLTNSK-YGPTPVAGNDIKSGIINFATGYNNLTAYKSSGIDEHTGI 103
Ct-SdbA         FT--SSTFPPGR-TVLKNNA-YGPIQIADNDPEKGILNFALAYSYIAGYKETGVAEESGI 103
Ac-ScaO         TN--STSVILGA-----------------NETSNG----INILYLDNSTTFSNPITSAGE 86
Cc-ScaO         EN--INGDDLAI-----------------HEAEDG----IRILYLDNS--LNNPIISSGT 84
Cc-ScaG         IT--DPDTNFAY-----------------HYSQDEN--LIRMLFLNETMQEDGVIRTEGV 88
Ac-ScaN         VS-----TTLSR-----------------NTNTDNG-----CMAFTDYIEGDKTITQSGV 82
                                                                           *

Ac-ScaC1        FASIKFKISETAPVGESYDITTNYSR--TSFYS--TGTEEIVNILCNNG--------- 131
Cc-ScaC1        FAAIKFKISEANPVGEVYDISVNYSR--TSFYS--TGTEEIKNVLYNDG--------- 131
Ac-ScaC3        FATINFKVSDSAKVGELYNITTNSAY--TSFYY--SGTDEIKNVVYNDG--------- 131
Ac-ScaC2        FAQVKFQISDTALQGETYKISQISSN--TSFYS--TAVTEITDVIYADG--------- 131
Cc-ScaC2        FAILKFKISDTALDGEVYKISTVLAN--TFFYS--TGVNEIKNVVYNDG--------- 131
Cc-ScaC3        IARIVFELLDP-KVGDVYWLLNYKDS--SGVYA--YGAKEITNVKYIDG--------- 138
Ac-ScaJ         IAQIQLKIKSTASDGIYN-LNYNLSR--CAVYA--SGTDEITGVIYNG---------- 129
Cc-ScaJ         MAYIKFTIADNASDGVSF-LTYNLSR--SAVFT--TGIDEIKGILYNNG--------- 130
Ac-ScaK1        VLNIEFRIKDDAPLGTYKIEAESLGM--GSFDSDSEYINRINDVYTSG---------- 127
Ac-ScaK2        ITEIGFRIKEDVPPGTYEIKAESLGM--GSFDG--EFINRITDVFSIG---------- 128
Ct-CipA4        FATIVAKVK------SGAPNGLSVIKFVEVGGFANNDLVEQKTQFFDGG--------- 133
Ct-CipA5        FATIVAKVK------SGAPNGLSVIKFVEVGGFANNDLVEQKTQFFDGG--------- 133
Ct-CipA6        FATIVAKVK------EGAPNGLSVIKFVEVGGFANNDLVEQKTQFFDGG--------- 133
Ct-CipA8        FATIVAKVK------EGAPNGLSVIKFVEVGGFANNDLVEQKTQFFDGG--------- 133
Ct-CipA3        FATIVAKVK------SGAPNGLSVIKFVEVGGFANNDLVEQRTQFFDGG--------- 133
Ct-CipA7        FATIVAKVK------SGAPNGLSVIKFVEVGGFANNDLVEQKTQFFDGG--------- 133
Ct-CipA2        FAKIRATVK------SSAPG---YITFDEVGGFADNDLVEQKVSFIDGG--------- 130
Ct-CipA9        FATIVAKVK------EGAPEGFSAIEISEFGAFADNDLVEVETDLINGG--------- 133
Ct-CipA1        FATIVATVK------SAAAAP---ITLLEVGAFADNDLVEISTTFVAGG--------- 130
Ct-OlpA         FFTIVMEVS------KDVKPGISPIKFESFGATADNDMNEMTPKLVEGK--------- 131
Ct-OlpC         FAKLTVRIKPDIFKDSGSTKKYSLITFGES-NFCDFDLKPILAVLKEG---------- 135
Ac-ScaA2        FAKVTFKVLNA-----G--GSSVGIT---GATFGDKNLGSVSATINAG---------- 124
Ac-ScaA3        FTKVTFKVLSS-----G--GSTVGIT---GATFGDKNLGNVSATINAG---------- 124
Ac-ScaA1        FANVTFKVLNS-----A--PTTVGIT---GATFGDKNLGNISATINAG---------- 124
Ac-ScaM1        FLNLTFKVLGS-----G--ATTVSIS---GATFGDKNLSTISSTLNSG---------- 124
Ac-ScaM2        FANITFKALSS-----G--TSTVSIS---GATFGDKNLNSVSAKFNSG---------- 124
Ac-ScaM3        FTNLTFKVLNS-----S--ATTVSIS---GATFGDKNLNTVSTTLIQG---------- 124
Cc-ScaM2        FANITFKALG---------TSTVTIS---KATFGDRTLKPMTAKLNAG---------- 122
Cc-ScaM3        FANITFKALG---------TSTVTIS---KATFGDRTLKPMTVKLNAG---------- 122
Cc-ScaM1        FANITFKVLG---------TSTVTIS---KATFGDRTLKPMTAKLNAG---------- 122
Cc-ScaMa1       FANITFKVSG---------TSTVTIS---KATFGDRSLKPVSATLTAG---------- 122
Cc-ScaMa2       FANITFKVSG---------TSTVTIS---KATFGDRSLKPVSATLTAG---------- 122
Cc-ScaMa3       FANITFKVSG---------TSTVTIS---KATFGDRSLKPVSATLTAG---------- 122
Cc-ScaMa4       FANITFKVSG---------TSTVTIS---KATFGDRSLKPVSATLTAG---------- 122
Cc-ScaMa5       FANITFKVSG---------TSTVTIS---KATFGDRSLKPVSATLTAG---------- 122
Cc-ScaMa6       FANITFKVSG---------TSTVTIS---KATFGDRSLKPVSATLTAG---------- 122
Cc-ScaMb2       FANITFKVSG---------TSTVTIS---KATFGDRSLKPVSATLTAG---------- 122
Cc-ScaMb3       FANITFKVSG---------TSTVTIS---KATFGDRSLKPVSATLTAG---------- 122
Cc-ScaMb4       FANITFKVSG---------TSTVTIS---KATFGDRSLKPVSATLTAG---------- 122
Cc-ScaMb5       FANITFKVSG---------TSTVTIS---KATFGDRSLKPVSATLTAG---------- 122
Cc-ScaMb1       FTNLTFKVISS-----ASTTATVTVN---KANFGDRNLSPITAQFSAG---------- 126
Ac-ScaA4        FANLTFKIKST-----AAAGTTAAVGIAGTATFGDSALKPITAVITDG---------- 129
Ac-ScaA5        FANLTFKIKST-----AVNGDVAAISKSGTATFGDKNLGPISAVIKDG---------- 129
Ac-ScaA6        FANLNFNIKSS-----AAIGSKAEVSISGTPTFGDSTLTPVVAKVTNG---------- 129
Ac-ScaA7        FANVTFKVIGT-----DG---LAAVN-AEDATFGDSSLSPVTASVVNG---------- 125
Ac-ScaD3        FVKLKFKVISS-----ARTSTSITVN---DASFGDKDLKSVRATLYSG---------- 126
Cc-ScaA3        FANLKFKVIT-------SAAVSTVVSIS-KATFADRQLKPITTGLING---------- 126
Cc-ScaA4        FANLKFKVIT-------SAAVSTVVSIS-KATFADRQLKPITTGFING---------- 126
Cc-ScaA2        FANLKFKVIT-------SAAVSTVVSIS-KATFADRQLKPITTDLIDG---------- 126
```

```
Cc-ScaA1          FANLQFKVIT-------SAAVSTTVSIS-KSTYANRSLAPVSANVIDG---------- 129
Cc-ScaA6          MLNLKFKINSN-----AKAGTSATVAIGDKPTFADRSLKPIKLAVVSG---------- 129
Cc-ScaA7          MLNLKFKINSN-----AKAGTSATVAIGDKPTFADRSLKAIKLAVVNG---------- 129
Cc-ScaA5          MINLKFKINSN-----AKAGTSATVAIGDKPTFADRSLKPIKLALVNG---------- 129
Cc-ScaA8          FAKLIFKVIG--------DEGLAEIKIE-KINFADRSLKPVSLESKNG---------- 125
Cc-ScaD3          FANIKFKVITS-----NRTTTTVEIN---KANFGDTSLKAIPVTLTDG---------- 126
Ac-ScaL2          VANLNFKVVGH-----TSGLTFIDLC---YATVADLNLNVIATELCSG---------- 126
Ac-ScaL3          VANLNFKVIGS-----SYEVTHINIS---NQTVGDINLYSVPTAVYPG---------- 126
Ac-ScaL1          FAELNFKINIS-----VSYQTSIKIY---DITVGDGNYASVPTKAIDG---------- 126
Cc-ScaHL1         FISPIFKKLQS-----YNESTSIKII---RSTFGDFNLNEVKAKIIQT---------- 126
Ac-ScaL4          FTNLTFKVLPT-------KCGITSINVVN-ATFGDIDLNNLNTTIVPG---------- 126
Ac-ScaG           FTKIKFKVKDT-----A-KVGENSIAISGDSTFGDTQLAAMKVEYKAG---------- 129
Cc-ScaHL2         FANITFKVLCK-----YDINTYISVR---SYTFKDGDLQDVYATPRST---------- 126
Ac-ScaI           LANLIFKITGN-----SDKTP-IKIN---NASFADRSFNILITTLNDG---------- 125
Ac-ScaH           FANLTFNVVSS-----SNVTAAVNAK---DVLIADGCINPVPTTIYPG---------- 126
Cc-ScaHL3         LANLTFRVLGS-----AEETT-VNIT---ESSVANRTLKRVNPIITPG---------- 123
Ac-ScaD1          IAIIGFKV-LQEKDTTISFADCTTMP---NGITGTILFDWDGSRIASG---------- 147
Cc-ScaD1          VAIIGFKV-IKAKDTTIHFQDTKSMP---NGIKGTSLYDWDG-KTLSG---------- 146
Ac-ScaB1          VAVVKFKV-LKEETSSISFEDTTSVP---NAIDGTVLFDWNGDRIQSG---------- 145
Cc-ScaB1          VAVIRFKV-LKEETTSIKFEDSTSLP---NGINGTLLFDWFGNRIDSG---------- 146
Ac-ScaE3          LAVISFKV-LTAKSTAVLFENSASMA---NGISGTMLFDWNGDKLLSG---------- 146
Cc-ScaE3          LATISFKV-LKAQSTSILFENSSSMP---NGISGTMLFDWYGNRILSG---------- 146
Ac-ScaE6          VAVINFKV-LAESATSVIFENSTSMP---NGINGTMLFDWYGDRIKSG---------- 146
Ac-ScaE7          IAVIQFKV-LASTATTLKFENSTSMP---NGIDGTMLFDWDGNRITSG---------- 146
Cc-ScaE6          LAVVTFKV-LKAGDTSIAFENTASMP---NAIDGTILFDWYGNKIKSG---------- 146
Cc-ScaE7          IAVIEFKV-LKEIETYVKFENSSSMP---NGINGTMLFDTDGNRITSG---------- 146
Ac-ScaE4          LAVIGFKV-LKQTATSITFENSGTMP---SAIIGTMLFDSYGNKINSG---------- 146
Cc-ScaE4          LAKISFKVTLSETLTYIRLENTGTMP---NAIDGTMLFDWYGNRINSG---------- 147
Ac-ScaE5          LAVISFKV-LKVQATSVSFASTNTMP---HGIDGTMLFDWDAKRIISG---------- 146
Cc-ScaE5          VATISFKV-LSKKITSIMFENTNTMP---NAIDGTILFDWNGERIKSG---------- 146
Ac-ScaE1          IAVIYFKV-LQEKVTSVVFENTKTMP---SAITGTNIFDWTGNKITSG---------- 146
Cc-ScaE1          VAVINFKV-ISDKPTSIVFEDTYRMP---GAVTGTMIFDWNGNRISSG---------- 146
Ac-ScaF           VAVIGFKV-LKAEETSIEFAKTSTMP---TAVSGVLLFDWDGNKLTSG---------- 147
Cc-ScaF           VAVIGFKV-RRAEETKIEFAQTSTMP---TAVSGVLLFDWDGNKLTSG---------- 147
Ac-ScaB2          LAVVGFKV-LQKKATTVVFEDSETMP---NGITGTTLFNWYGNRIQSG---------- 147
Ac-ScaB3          VAVVGFKA-LQKKATTVVFEHSVTMP---NGIIGTTLFNWYGNRITSG---------- 147
Cc-ScaB2          LAIIGFKV-LQKKPTTVLFEDTEVLP---NGINGTTLLNWNGDRIQSG---------- 147
Cc-ScaB3          IAVIGFKV-LQKKETAVLFENAASIP---NAIDGTVLIDWYGNKISSG---------- 147
Cc-ScaB4          IAVIGFKV-LQKKETAVLFENAASIP---NAIDGTVLIDWYGNKISSG---------- 147
Ac-ScaB4          VAVVGFKV-IKAKNTAITFEDCDSMP---SGITGTMLFNWDGEIIKSG---------- 147
Cc-ScaB5          ILVIGFEV-LQAKNTFIKFEDSTSMP---NGIEGTILFDWNGDRIADG---------- 147
Ac-ScaD2          LGVVGFKV-LKEGTTTVKFENADSIS---NGISGTLLSDWYGNRIYSG---------- 147
Cc-ScaD2          LAVVGFKA-IKEGNAYVYFENTNSMP---NAITGTMLVDWNNNVIKSG---------- 147
Ac-ScaE2          LAVISFKV-LQGKATTVSFEDSRTMP---NGITGTILCNWNAERISG----------- 145
Cc-ScaE2          LGVIEFRV-LDAKATAVSFEDSKYMP---NSYSGTLLFDWNAEKISG----------- 145
Ac-ScaP           LAVIKFKV-INSKQVQIKFSVSDAIP---ESNTGVNLIDWDGSKIIG----------- 145
Ct-Olpb2          IGKIGFRV-LKAGSTAIRFEDTTAMP---GAIEGTYMFDWYGENIKG----------- 147
Ct-Olpb3          IGKIGFRV-LKAGSTAIRFEDTTAMP---GAIEGTYMFDWYGENIKG----------- 147
Ct-Olpb4          IGNIGFRV-LKAEDTTIRFEELESMP---GSIDGTYMLDWYLNRISG----------- 147
Ct-Orf2p1         IGKIGFKA-LKLGKTEIKFENTPVMP---GAKEGTLLFDWDAETITE----------- 148
Ct-Orf2p2         IGKVGFKV-LKAANTTVKLEETRFTP---NSIDGTLVIDWYGQQIVG----------- 146
Ct-Olpb1          IGEIGFKV-LKKQNTSIRFEDTLSMP---GAISGTSLFDWDAETITG----------- 146
Ct-SdbA           IAKIGFKI-LQKKSTAVKFQDTLSMP---GAISGTQLFDWDGEVITG----------- 146
Ac-ScaO           LIKLKFTVNTATTDGAFELTYNDGWS--FGKRINSDVDDISNVVFKPG---------- 132
Cc-ScaO           LVNLKFQINSNVTDGTCILGYNDNWS--FASFTDSKFYIYSQVKFKAG---------- 130
Cc-ScaG           LATVRLKVKDN------AKSSESELAIVGKATFADTKLSTLDIELSTGD--------- 131
Ac-ScaN           FCKMTFKANTTCPAGKYSFKVLTNDTIRKTSFVKDDTFTNVLVNSQDSVVDVVLHHSG 140
                  .
```

Figure S1: Multiple sequence alignment of 106 cohesin sequences originated from the genomes of *C. clariflavum* (Cc), *A. cellulolyticus* (Ac) and *C. thermocellum* (Ct). Alignment length: 148; Identity (*): 1 residue = 0.67%; Strongly similar (:): 3 residues = 2.03%; Weakly similar (.): 3 residues = 2.03%; Different: 141 residues 95.27%. All the accession numbers for *C. clariflavum* cohesin-containing proteins can be found in Figure 1, and th accession numbers of *A. cellulolyticus* and *C. thermocellum* cohesin-containing proteins can be found in Dassa B, Borovok I, Lamed R, Henrissat B, Coutinho P, Hemme CL, Huang Y, Zhou Z, Bayer EA: **Genome-wide analysi of *Acetivibrio cellulolyticus* provides a blueprint of an elaborate cellulosome system**. *BMC Genomics* 2012, **13**:210.
