# Supplementary material for: Cellulosomics of the cellulolytic thermophile Clostridium clariflavum
Source: Biotechnol Biofuels. 2014 Jul 1;7:100. doi: 10.1186/1754-6834-7-100 (PMC4582956; doi:10.1186/1754-6834-7-100)
Supplement: Additional file 2: Figure S2 — Multiple sequence alignment of the C. clariflavum 74 dockerin modules. Cyan highlight indicates putative calcium-binding residues. Yellow highlight indicates putative recognition residues. Gray highlight marks the last C-terminal residue of a corresponding protein. x indicates a computational fusion of Clocl_2272 [YP_005046783] and Clocl_2271 [YP_005046782] to reconstruct a complete dockerin motif (a stop codon TAA of Clocl_2272 was replaced with NNN). BIL, bacterial intein-like domain; CARDB, cell adhesion-related domain found in bacteria; CBM, carbohydrate binding module (followed by family number); CE, carbohydrate esterase (followed by family number); COH, cohesin; DOC, dockerin; EXPN, expansin; FN3, fibronectin type III domain; GH, glycoside hydrolase (followed by family number); LNK, linker; PL, polysaccharide lyase (followed by family number); Serpin, serine protease inhibitor; SIGN, signal peptide; UNK, unknown region; X, X domain. Alignment length: 84. Identity (*): 5 identical residues = 5.62%. Strongly similar (:): 1 residue = 1.12%. Weakly similar (.): 3 residues = 3.37%. Different: 80 residues = 89.89%. [file 1754-6834-7-100-S2.pdf]

Gene	Alignment	Annotation
	10 20 30 40 50 60 70 80	
Clocl_1157	MSETNKGLVGDVDGNGEINSIDYAYMKMVLLGIRKDFPIED-----GLWAADVNGDGNFNSIDCAYMKMYLLGRIKEFPKKNIV	SIGN-DOC-UNK-CBM32-UNK-
Clocl_3208	EFPEDKPLIGDVDGNGDVNSIDYAFMKMYLLGIINDFNVED-----DLWASDVNGDGVFNSIDYAFMKLFLLGRIKEFPKQNLI	-UNK-DOC-UNK-
Clocl_1587	TSYSEERLVGDIDGNGVFNSIDYAKLKALMLGYKIELPED------YEWAADVDGSGMINSIDIAYMKSYLLGKIKIFPKERPT	UNK-COH1-UNK-DOC-FN3-FN3-CARDB- CARDB-CARDB-UNK-BIL
Clocl_1863	YNPVPTIKFGDVNCDGNIDSIDYALMKSYLLGIINKFPVEY-----GLLAADLNGDGDFNSIDFAILKSYLLGIIKVFPAEDIS	SIGN-UNK-GH81-DOC-
Clocl_2488	ANEIKDIVYGDLNGDGNADSIDYATLKMYLVGIIQLSEK-------QLKASDLNLDGDVNSIDFAHFKRYLLGMIKSLPVEPVE	SIGN-DOC-LNK-PL9_1
Clocl_0088	PTPAQSVLCGDVNDDGQRNAIDYALIKTYLLGIIKEFPSPK-----GLTAADVNGDTRVDSIDFAMYKQFLLGMIKVFPAEAM-	SIGN-GH43_E-CBM42-DOC
Clocl_1564	DTTEPEVLVGDVNKDGDINVIDYALMKKYVLGMLDEI---------DVAAADINVDGEVNSIDCSLMKAYLLGMIKEIPYYEET	SIGN-GH44-DOC-X3-CBM44
Clocl_0311	SGEIGSMILGDVNNDGDVNSIDFAYMKMNLLGIKGSLLNDE-----NIFVADLNGDGKFDSIDLAIMKGYLLGKIKEFPIGQSI	SIGN-UNK-DOC-UNK-
Clocl_0467	PTPVENILYGDVNGDNEVNSIDFAYMKMYLLGITNKLPM-------DAKYGDLNGDGYVDSIDLALIKMYLLGYIKKFPVESMN	SIGN-UNK-DOC-
Clocl_2271x	MSVGAASLYGDLNxDDSVDSIDFAIMKGTLLSKLTIG---------NITLADLNGDGYINSLDLALLKMYLLGKISKFPIEDLN	
Clocl_4124	VNVSAGSLKGDVNSDNSIDSIDYAVMKSYLLGISSQIPF-------DVSVADLNGDGSVNSIDLALMKSYLLGIITSFPAETSN	SIGN-UNK-DOC-UNK-X135-UNK-pfam08757-UNK-
Clocl_1051	FITSYAAVRGDLNNDNRVDSLDYAFYKMYLLGSYHLS---------DISVADLNGDGDTNSIDYAILKQYLLGMIKEFPADANP	SIGN-DOC-UNK-
Clocl_2096	IVNSEPVKRGDINSDGDINSIDFALYKLYLLGGYQIN---------DITVADLNGDGSANSIDYGYLKLYLLGRISTFPADEIS	SIGN-DOC-UNK-X187
Clocl_2437	TPTTVKTKLGDINGDGDINSLDFAEFKMYLLGMTD-----------KISNADLNGDNDVNSIDYALLKQYLLGMISVFPAEA--	SIGN-GH43-CBM6_2-LNK-DOC-
Clocl_1975	TPTSNTYLVGDVNHDNNINSIDYALMKSYLLGMKLPENTF------FESEADVNGDGDINSVDYALLKQRLLGMISKFHVEE--	SIGN-GH9_3-LNK-DOC-
Clocl_0355	RKYLPDRVVGDLNLDGEINSVDCVIMKAYLLGIYTNLENDKE----RFGAADINSDGNVNSIDYAILRKYLLGMGELPTPSPKP	SIGN-UNK-DOC-UNK-
Clocl_1556	KPIVDNFILGDINGDGYIDSNDYGMLKLYLLGFIKDFSYKH-----GFQAADVNSDGNVNSIDFAIFKRYLLGMITDFPQTPIP	SIGN-UNK-DOC-cd07761
Clocl_1566	EPVKTTYTLGDLDNNGSIDSIDYAMLKMYLLGLTKFDEE-------TYNRADVNRDTNVNSIDCALIKMYILGMIKSF------	SIGN-GH9-UNK-CBM3_1-UNK-CBM3_2-DOC-
Clocl_3327	PTGTAPQIMGDIDGDGSVTSIDFALLKKYLLSGIPDLS--------NVWYADVNSDGSVNSIDYAFIKAYLLGMIKEFPGSAQP	SIGN-X70-CE12-UNK-DOC-UNK-CBM35-UNK-CE12
Clocl_1480	PINPGYILLGDVDQNGTINSLDYAKYKMYLLGMISS----------LPEEGDINRDGDMNSIDYAMLKQHLLGIINLEELSGPK	SIGN-GH11_6-LNK-CBM6_2-DOC-CE6
Clocl_3361	NGGNTGGLLGDVNNSGTIDSIDLAIYKQYLLGMISSFP--------APQNADIDNDGNYTSIDFAMIKQHLLGMIDLTTRGNPN	SIGN-GH11_6-UNK-CBM6_2-LNK-GH11_6-LNK-DOC-LNK-CBM6_2-LNK-CE4
Clocl_1893	TRLVITIYLGDVDENKVVNSLDLAKYKQYLLGEIKS----------LPEAGDVNQDGEMNSIDYALIKAYLLGLVKDLGTKTIW	-UNK-DOC-UNK-CE3
Clocl_3356	PRIPRGTVFGDINNSDTLDSFDLALFKAYLLGMAEIEPDPQ-----KKYIWDANADGNINSIDYAMLKRYFLGILIELPIQMCV	SIGN-DOC-DOC-DOC-
Clocl_2489	ETANSKYAVGDINQDGTVNSLDYAMLKGYLLGMKEFSDIGQ-----RILQADVNGDGCIDSLDFASFKLFLLKGIEFPSNRKIN	SIGN-DOC-UNK-
Clocl_0710	MVRSTPYIYGDLNGDKLVNSIDFALLKIYLLGYSKEFPYEY-----GIKSADLNRNGEVDSIDFAILRSFLLGIIKAIPVGAT-	UNK-CBM35-DOC
Clocl_4012	KPTNNEFVYGDINGDKRVDSIDYALLKSYLLGFSSEFPYVN-----GKKAADVNCDGNVDSIDFALLKGYLLGIFNYLPVGV--	SIGN-GH53-UNK-DOC-
Clocl_1811	TPTPQSVVIGDLNYDNRVDSIDYAYLKMYLLGQIYDFPTPY-----DMQAADLNGDGKIDSIDYAYLKMYLLGFINKFPVQ---	SIGN-GH16_lic-LNK-DOC
Clocl_1824	TPINEDVIYGDLNGDKNIDSIDYAYLKMYLLGMIKEFPSDN-----GLKAADLNGDGSINSIDYAYLKSYLLGIIKVFPVNEM-	SIGN-CBM35-UNK-GH26_1-UNK-DOC-
Clocl_3253	TPTNPNIVYGDLDGNKEVNSVDYALLKGVLLGIIRTDV--------DMAAADLNLDGDVNSIDYALLKMYLLGQIKELPYRLP-	SIGN-GH9_3-CBM3_1-UNK-DOC-
Clocl_3255	TPTNPQIKYGDLNNDNEINSIDYAYLKQYLLGMSTQNI--------NLAAADVNLDGDINSIDFALVKKYLLGMINTLPYIN--	SIGN-GH9_3-UNK-DOC-
Clocl_3058	IPDPINFIYGDLNGDKAVNAIDYALMKRILLGMSGELLSDD-----WEKAADLNMDGSINSIDFALLKKYLLGMLKTLPYTNTA	SIGN-UNK-CBM42-UNK-GH43_G-UNK-DOC-
Clocl_3968	IPVFSESLFGDLNGDNSVDSIDYALLKTYLLYTRAPSGDN------WMEHADVYRDGEINSLDLAILKKYLLGMTKSLPFNPEG	SIGN-DOC-UNK-SERPIN
Clocl_0906	TPTPKPSMKGDITLDGEINSIDYATLKMHLLGITTLTPE-------QLANADINDDNDVNSIDYALLKSYLLGIIKEF------	SIGN-GH74-LNK-DOC-
Clocl_1981	TPTPSNVIKGDINGDGEINSIDYAILKSYLLGLTKNLPAED-----ELAVADLNGDKEINSIDYALLKMYLLGMIKDFS-----	SIGN-GH5_1-LNK-DOC-
Clocl_1055	PSNGGTVIKGDVNGDGEVNSIDYALMKSKILGLIYNFPYIY-----GDLAADMNDDGDFNSLDYALLKMKLLGLTSI-------	SIGN-GH8-LNK-DOC-
Clocl_2763	TPPHYTVLKGDVNGDGEVNSIDYGYVKMYLLGMINKFPTADPNV--GYAGADIDGNGDVNSIDFALLKSILLGIKSND------	SIGN-UNK-GH43_F-CBM13-LNK-DOC-
Clocl_2097	PTPIGNVKKGDINGDGEVNSIDYALLKRYLVGMDTTLTPN------SYAAADINGDGDINSLDYANLKLILLGMNN--------	SIGN-CE3-UNK-CE3-DOC

```
Clocl_2438  PTPSFNVKKGDVNGDGDVNSIDFAYLKRYLLGMDSSFPSPY-----GSIAADLNNDGRIDSIDYAHLKLILLGMYYEN------  SIGN-CE3-DOC-
Clocl_3239  PTPASTILKGDINGDGEINSIDFAYLKKYLLGMNVPFTN-------ILETADLNNDNSVDSIDYALLKCLILGIK---------  SIGN-UNK-GH18-UNK-DOC-
Clocl_2083  SQILPQIVEGDTNGDGEFNSIDYATLKQILLGINTENRYIY-----WEQASDLDKNGTVDSIDYALMKMRLLGIK---------  SIGN-GH11_6-UNK-GH10-DOC-
Clocl_3932  TPTPSNYLLGDVNGDNEFNSVDYALLKMYLLGMGTPDYPL------WKEAADMNKDGRIDSIDYALMKKTLLGIKD--------  SIGN-GH5_1-UNK-DOC-
Clocl_0901  TPTPQKRIVGDLNSDGKFDSIDIALLKSHILGIETFNI--------DLTYADVNADGDINSIDYAYMKQRILGMISKFPIE---  SIGN-CBM35-UNK-GH26_1-UNK-DOC
Clocl_1061  TGLPGKGIIGDANWDGDFNSIDIGLVKMYLLGMINENGI-------NVKACDVNADGEVNSIDFAFMKQKMLGMIDKFPAE---  SIGN-UNK-X140-UNK-X139-UNK-DOC
Clocl_1551  SDVPSKIVYGDVNGDGKFDSIDCATVKMYLLGMIEGFTYSE-----GFKAADVNGDENINSIDFALMKSRLLGIINKFPVEN--  SIGN-CE*_-CE6-DOC-
Clocl_2046  AISTSKAVIGDLNGDFKFDSIDCALMKMYLLGMIKSFEVED-----ELYAADVNGDTLINSVDFAYMKKMLLGMITEFPKKSME  SIGN-UNK-DOC-UNK-COG5184-UNK-
Clocl_1056  TATPVDFVKGDLNGDKTFNSIDYAYLKMHLLGMNKLNEA-------QLLAADVDNNGQVDSIDYAIMKQVLLGIRKDF------  SIGN-GH9-UNK-CBM3_1-LNK-DOC-
Clocl_2225  TPTNNEVMKGDLDGDKDITSIDYAYLKMHLLGMSKLNEQ-------QLKAADVDSNNSVDSIDLALIKAYLLGIIKSF------  SIGN-GH9- CBM3-DOC-
Clocl_3917  NPGGEDIVLGDINFDGDINSIDYALLKAHLLGINKLSGD-------ALKAADVDKNGDVNSIDYAKMKQYLLGISKEF------  SIGN-UNK-CBM4-UNK-X229-GH9_1-UNK-DOC-
Clocl_4007  GGETGDIKLGDINFDGDINSIDYALLKAHLLGINKLSGD-------ALKAADVDQNGDVNSIDYAKMKSYLLGISKDF------  SIGN-GH48-UNK-DOC-
Clocl_1122  VSSPVTLVYGDLNGDDDFNSIDFGLLKLVLLGLKSRTEI-------NEKAADVDGNGHIDSIDYALMKQRLLGIIKKFPVEN--  SIGN-GH5_1-UNK-DOC-
Clocl_2712  ISMKSKVIKGDINGDGVFNSIDLALMKMYLTGSIKFTEE-------QFEAADVDNSGEVNSIDYAIMKQVLLGYRPGF------  -UNK-DOC-
Clocl_0923  AADDVSAMYGDLNGDGSVNSIDYAIMKSYLIGIRSNFPVLN-----GEVVGDVNADNSVNSIDYAFIKSYLLGLISEFPAEKTR  SIGN-DOC-UNK-CBM35-UNK-CE8
Clocl_4161  GSQMISTKYGDLNGDGERDSIDLALIRAYLIGLISDFPVSN-----GKIAADVNGDNDVNSIDYAYMKSFLLGFISEFPAENNP  SIGN-DOC-UNK-CBM35-UNK-PL11_1
Clocl_2441  SGNPGSRKYGDLDLDGQVTAIDLATFKSHLLGMSTLTGE-------ALANADVSGDGDVNSIDMAIIKQYLLGMISKFPAENNT  SIGN-GH11_6-UNK-CBM6_2-UNK-DOC-UNK-GH10
Clocl_2447  GGTNTTIGLGDLDGDGYVTSVDLALLKRHLLGMGYLTGE-------SLAKADVNRDGDVNSLDFAYLKAAILGIIVDSPDL---  SIGN-CE1-UNK-CBM6_2-DOC-
Clocl_1869  SMRAAELMYGDLNDSKSVDSIDYAIMKSYLLGMRSLTGD-------ALTAADVNGDGSVNSIDYAIMKQYLLGIISVFPASEQV  SIGN-DOC-GH43
Clocl_0350  PTPTTSYVYGDVDGNDIVNSIDYAYIKQYLLGMITEFPGAN-----GMHNADVDGSGSINSIDFAYVKQYLLGMISKFPAEENA  SIGN-GH5_4-LNK-DOC-LNK-CE2
Clocl_3029  SKQTVGFILGDVDGNGKIDSIDFATLKQYMLGMIKTLPSPY-----EEIAADVDGNGTINVIDLAYLKKYLLGMISKFPAEVN-  SIGN-CBM30-X229-GH9-DOC-
Clocl_1795  DTESASIIYGDVNGDGDVNSIDYGYMKWYLLGQINSFPVDN-----GDKVADLDGDGRITSIDCAYMKMYLLGMIQKFPVEQ--  SIGN-GH30-X92-DOC-
Clocl_4034  TKNEPTIKKGDVDGDGNITSIDLAYYKKYLLGQINDFPVEN-----DMQVADLDGNGAIDSIDLAYLKSYLLGMITKFPASN--  SIGN-CE1-DOC-
Clocl_3147  ETEHNNVLIGDVNGDNDVNSIDLLYMKKYILGMVDTFPVET-----GFYCADVDGNNQIDAIDLAYMKKYLLGMIKDFPKSADS  SIGN-UNK-DOC-UNK-
Clocl_2746  ENPVPEVIYGDVNGDGDFNSIDYGYLKMYLLGQIKEFPSKD-----GMIAADVNGDGTVNSIDYAYMKMRLLGQISKFPVE---  SIGN-GH30-UNK-CBM6_2-DOC-
Clocl_3132  PTPSSAVIYGDVNQDGTFNSIDYGFLKMYLLGMVKEDSI-------NKQAADVDGNGTIDSIDFAYMKMHLLGMINKFPVQENI  SIGN-GH26-LNK-DOC-
Clocl_1047  SVFAESIIYGDINGDGEVNSIDYAILKKYLLGKIKEFDKPN-----AIKAADVDGNEEINSIDFAFMKKYLLGLIKVFPAYEKS  SIGN-UNK-DOC-UNK-
Clocl_2354  SVQTTKVIYGDINGDSYCNSIDLAIIRSYLLGKIKSFDDIAPSGYDALKAADVNGDGEINSIDYAFMRKYILGIIREFPAESKE  SIGN-UNK-DOC-LNK-X72-X72-X72-X72-FN3-FN3-UNK-X83-UNK-
Clocl_1864  PTPTPNVLYGDINGDKDINSIDLAILKQYLLGMIKKFDV-------PDEVADLNGDGEINSIDYAYFKMYILGMIKEFPVNAR-  SIGN-GH9-LNK-CBM3_1-LNK-CBM3_2-LNK-DOC-
Clocl_1567  TPEPTKYIYGDVNGDGDINSVDYALIKMYLLGMIKEFEYEY-----GSKAADVDGNGVINSIDYAYMKMYLLGMIKEFPVEEK-  SIGN-GH9_3-UNK-CBM3_1-UNK-DOC-
Clocl_3001  TPTPNDYIFGDVNGDKEVNSIDFAIMKQFLLGMIKEFPYEH-----GAKAGDLNGDGNINSIDYALLKQYILGIIKEFPIEQ--  SIGN-GH9-UNK-CBM3_1-UNK-CBM3_2-UNK-DOC-
Clocl_1806  PTPVTKVIFGDLNGDETVNSIDYAYMKSYLLGMMKEFPSEN-----GLIAADVNGDGDINSVDYALMKMYLLGMIKEFPAASK-  SIGN-GH9-CBM3_1-LNK-DOC-
Clocl_1298  FISSAQLQVGDVNGDNNVDSIDFALMKSFILKIINTLPAED-----SLLAGDLDGDGSINSIDCALMKQYLLGMIKVFPKTQSP  SIGN-DOC-LNK-EXPN-CBM63
Clocl_1862  FSLEGQVTVGDINGDGYVDSIDYANLKMYVLGLIKDFPTAE-----GSWAADVDGNNSIDSIDCALMKSYLLGIIKKFPKSDSL  SIGN-DOC-UNK-EXPN-CBM63
Clocl_3305  EAPSHKFIYGDVDGNESVRINDAVLVRDYVLGKIDEFPYEY-----GMLAADVDGDGNIRINDSVLIRDFVLGKISLFPVEEQ-  SIGN-COH1-COH1-LNK-COH1-LNK-COH1-LNK-COH1-UNK-DOC-
Clocl_3920  ADTGSNFVYGDVNGDGSVDSLDLIILQKYVLGNIDKFPSEN-----GVKAADLDVNGVVDSIDYELFKQYLTGKIKDFPVNNSP  SIGN-UNK-DOC-UNK-
Clocl_2276  FAYCAEFVYGDVNGSGSVDSVDYALVKGYILGMITEFPGPD-----GKKAGDVNADGEINSIDFALMKSYLLGLIKKFPAEDIP  -UNK-DOC-UNK-X188
                     **   .      *    :  .                     *   .      *
```

Figure S2: **Multiple sequence alignment of the *C. clariflavum* 74 dockerin modules.**

Cyan highlight indicates putative calcium-binding residues
Yellow highlight indicates putative recognition residues
Grey highlight marks the last C-terminal residue of a corresponding protein.
x indicates a computational fusion of Clocl_2272 (gi|374296592) and Clocl_2271 (gi|374296591) to reconstruct a complete dockerin motif (a stop codon TAA of Clocl_2272 was replaced with NNN).

Abbreviations:

**BIL**	Bacterial intein-like domain
**CARDB**	Cell adhesion-related domain found in bacteria
**CBM**	Carbohydrate binding module (followed by family number)
**CE**	Carbohydrate esterase (followed by family number)
**COH**	Cohesin
**DOC**	Dockerin
**EXPN**	Expansin
**FN3**	Fibronectin type III domain
**GH**	Glycoside hydrolase (followed by family number)
**LNK**	Linker
**PL**	Polysaccharide lyase (followed by family number)
**Serpin**	Serine protease inhibitor
**SIGN**	Signal peptide
**UNK**	Unknown region
**X**	X domain

Alignment length: 84. Identity (*): 5 identical residues = 5.62 %. Strongly similar (:): 1 resiue = 1.12 %. Weakly similar (.): 3 residues = 3.37 %. Different: 80 residues = 89.89 %.
