# Supplementary material for: Cellulosomics of the cellulolytic thermophile Clostridium clariflavum
Source: Biotechnol Biofuels. 2014 Jul 1;7:100. doi: 10.1186/1754-6834-7-100 (PMC4582956; doi:10.1186/1754-6834-7-100)
Supplement: Additional file 3: Table S1 — List of primers for the C. clariflavum cohesin and dockerin modules that were cloned in this study. Nucleotides shown in bold indicate restriction sites added to the primers. [file 1754-6834-7-100-S3.pdf]

Table S1: List of primers for the *C. clariflavum* cohesin and dockerin modules that were cloned in this study. Nucleotides shown in bold indicate restriction sites added to the primers.

Construct	Primer name	Nucleotide sequence
CBM-Coh A1	U-BamHI-ScaA1	5'-AATATA**GGATCC**GCACAAGATGATTCGACGGC-3'
	L-XhoI-ScaA1	5'-ATATTA**CTCGAG**TCAAGCGTTTGTTGGTGTTG-3'
CBM-Coh A5	U-BamHI-ScaA5	5'-AATAAA**GGATCC**AATGCTTTGACAGTAGCAGT-3'
	L-XhoI-ScaA5	5'-TATATA**CTCGAG**TCATGTTACAGGTGTTGTTC-3'
CBM-Coh A8	U-BamHI-ScaA8	5'-ATATTA**GGATCC**GACTTAGAAGGATTCAATGTTGTAATAG-3'
	L-XhoI-ScaA8	5'-TATAAT**CTCGAG**TCAAACTGTAAATCCTTCAACTATAGTTCC-3'
CBM-Coh B4	U-BamHI-ScaB4	5'-ATAATA**GGATCC**GCAGTACCAACAACCGATAG-3'
	L-XhoI-ScaB4	5'-AATATA**CTCGAG**TCAAGATATTACTTCAGCAT-3'
CBM-Coh B5	U-BamHI-ScaB5	5'-AATATA**GGATCC**ATAAGAGACAGCTATATTAC-3'
	L-XhoI-ScaB5	5'-TATAAT**CTCGAG**TCATGCTTCATTAACTGTTA-3'
CBM-Coh C1	U-BamHI-ScaC1	5'-AATATT**GGATCC**GCAGGGCAATTACAAATTGA-3'
	L-XhoI-ScaC1	5'-TATTAA**CTCGAG**TCAATTTGAGCCAACCAATA-3'
CBM-Coh C4	U-BamHI-ScaC4	5'-ATTATA**GGATCC**GGTACACCAATGAATGTGAA-3'
	L-XhoI-ScaC4	5'-TTATAT**CTCGAG**TCATGCTTCTAAAATTGTTA-3'
CBM-Coh D1	U-BamHI-ScaD1	5'-AATTTA**GGATCC**AGTAGTGACTCGTTTATCGA-3'
	L-XhoI-ScaD1	5'-TAATTA**CTCGAG**TCATTGCTTCACTTCATATC-3'
CBM-Coh D2	U-BamHI-ScaD2	5'-AATATA**GGATCC**AATGGCTACATATCAATTGA-3'
	L-XhoI-ScaD2	5'-ATATTA**CTCGAG**TCATGCTTTTGATACTTTAA-3'
CBM-Coh D3	U-BamHI-ScaD3	5'-ATTATA**GGATCC**GCAACGTCAACTGGGTTTGA-3'
	L-XhoI-ScaD3	5'-ATATTA**CTCGAG**TCATGTTACATAATTAAGTC-3'
CBM-Coh E1	U-BamHI-ScaE1	5'-AATATA**GGATCC**GATACTCAGAATTCAAGTATAG-3'
	L-XhoI-ScaE1	5'-TTATAT**CTCGAG**TCAATTGTCAGAGTTGATTAATCC-3'
CBM-Coh E7	U-BamHI-ScaE7	5'-AATATT**GGATCC**GCTTTGTCCCGCTACATTAC-3'
	L-XhoI-ScaE7	5'-TTATAA**CTCGAG**TTAGTTTATTTTTTCAGGTTG-3'
CBM-Coh F	U-BamHI-ScaF	5'-TTATAT**GGATCC**GATACCGCACCAAGTATCAAAATTG-3'
	L-XhoI-ScaF	5'-ATATTA**CTCGAG**TCAATTTGAAGTTGCATTTATAGC-3'
CBM-Coh G	U-BamHI-ScaG	5'-AATTAA**GGATCC**ACAGATACAACTAAGACCGT-3'
	L-XhoI-ScaG	5'-TTATAA**CTCGAG**TTATTTAACAACTGTTACATC-3'
CBM-Coh H/L1	U-BamHI-ScaH/L1	5'-AATATT**GGATCC**CAAAAAGATTTTACCGTTGAAATTGC-3'
	L-XhoI-ScaH/L1	5'-TTATAT**CTCGAG**TCAAGGGATATTGTCAGCGGTTTGAA-3'
CBM-Coh H/L2	U-BamHI-ScaH/L2	5'-AATATT**GGATCC**ATCTTTCAATTTGCTATAGG-3'
	L-XhoI-ScaH/L2	5'-TTATAT**CTCGAG**TCATGGAAGCTCGATTCCCG-3'
CBM-Coh H/L3	U-BamHI-ScaH/L3	5'-TTATTA**GGATCC**AGCTTTGGTATTGAAATAGG-3'
	L-XhoI-ScaH/L3	5'-ATATTA**CTCGAG**TCATCCCGAAGAGGGTAAAAGG-3'
CBM-Coh J	U-BamHI-ScaJ	5'-TTATAA**GGATCC**GCCGATGATTCAATGCAGGT-3'
	L-XhoI-ScaJ	5'-AATATA**CTCGAG**TCATATCGCAGCCTTTCCGA-3'
CBM-Coh O	U-BamHI-ScaO	5'-ATATTA**GGATCC**AGTCAAACTGATAACTATAC-3'
	L-XhoI-ScaO	5'-TTATAA**CTCGAG**TCACACCATAATAGGCTGAT-3'
Xyn-X-Doc A	U-KpnI-ScaA-XDoc	5'-ATATTA**GGTACC**TTTTACAGTTTCAGGTTATATAGC-3'
	L-BamHI-ScaA-XDoc	5'-ATTTAA**GGATCC**TTAATAGTCTGAAGATACTTT-3'
XynDoc B	U-KpnI-ScaB-Doc	5'-AATTAT**GGTACC**TTCTCATAAATTTATCTATGG-3'
	L-BamHI-ScaB-Doc	5'-ATATTA**GGATCC**CTATTGTTCTTCAACTGGGA-3'
Xyn-X-Doc H/L	U-KpnI-ScaH/L-XDoc	5'-TTATAA**GGTACC**TTTACCCTCTTCGGGATATAAAG-3'
	L-BamHI-ScaH/L-XDoc	5'-AATATT**GGATCC**TTAATAATCTGCAGAAGTCTTG-3'
XynDocGH48	U-KpnI-GH48-Doc	5'-AATATT**GGTACC**TGAAACTGGAGATATTAAAC-3'
	L-BamHI-GH48-Doc	5'-TTATAT**GGATCC**TTAAAAATCTTTGCTTATTC-3'
